# A new human-based metaheuristic algorithm for solving optimization problems based on preschool education

**DOI:** 10.1038/s41598-023-48462-1

**Published:** 2023-12-06

**Authors:** Pavel Trojovský

**Affiliations:** Department of Mathematics, Faculty of Science, University of Hradec Králové, Rokitanského 62, 500 03 Hradec Králové, Czech Republic

**Keywords:** Engineering, Mathematics and computing

## Abstract

In this paper, with motivation from the No Free Lunch theorem, a new human-based metaheuristic algorithm named Preschool Education Optimization Algorithm (PEOA) is introduced for solving optimization problems. Human activities in the preschool education process are the fundamental inspiration in the design of PEOA. Hence, PEOA is mathematically modeled in three phases: (i) the gradual growth of the preschool teacher's educational influence, (ii) individual knowledge development guided by the teacher, and (iii) individual increase of knowledge and self-awareness. The PEOA's performance in optimization is evaluated using fifty-two standard benchmark functions encompassing unimodal, high-dimensional multimodal, and fixed-dimensional multimodal types, as well as the CEC 2017 test suite. The optimization results show that PEOA has a high ability in exploration–exploitation and can balance them during the search process. To provide a comprehensive analysis, the performance of PEOA is compared against ten well-known metaheuristic algorithms. The simulation results show that the proposed PEOA approach performs better than competing algorithms by providing effective solutions for the benchmark functions and overall ranking as the first-best optimizer. Presenting a statistical analysis of the Wilcoxon signed-rank test shows that PEOA has significant statistical superiority in competition with compared algorithms. Furthermore, the implementation of PEOA in solving twenty-two optimization problems from the CEC 2011 test suite and four engineering design problems illustrates its efficacy in real-world optimization applications.

## Introduction

An optimization problem involves finding the best solution from multiple possible solutions, typically defined by decision variables, constraints, and an objective function^1^. Optimization aims to identify the optimal solution for the given problem among all possible alternatives^2^. Optimization techniques can be classified into deterministic and stochastic approaches. Deterministic approaches, which can be categorized into gradient-based and non-gradient-based methods, are effective for solving linear, convex, differentiable, and continuous optimization problems^3^. However, as science, engineering, technology, and industry progress, numerous real-world optimization problems arise that exhibit nonlinear, nonconvex, non-differentiable, discontinuous, and high-dimensional characteristics. Deterministic approaches are inadequate for solving such problems as they struggle to navigate the solution space efficiently and often become trapped in suboptimal solutions. Consequently, researchers have introduced stochastic approaches, known as metaheuristic algorithms, to address these limitations in optimization^4^.

Metaheuristic algorithms have gained significant popularity due to their ability to provide satisfactory solutions for optimization problems through random search, without relying on gradient information. These algorithms offer several advantages, including conceptual simplicity, easy implementation, problem-type independence, effectiveness in handling non-linear, non-convex, non-differentiable, discontinuous, high-dimensional, and NP-hard problems, as well as efficiency in exploring unknown search spaces^5^.

To effectively solve optimization problems, metaheuristic algorithms must demonstrate proficiency in global and local search processes. Global search, known as exploration, involves thoroughly exploring all regions of the search space to uncover the primary optimal region. Local search, referred to as exploitation, pertains to the algorithm's ability to converge towards potentially improved solutions near already identified promising solutions. Alongside exploration and exploitation, achieving the desired performance of metaheuristic algorithms relies on striking a balance between these two aspects during the search process^6^.

Due to the random nature of their search process, metaheuristic algorithms do not guarantee finding the global optimum for optimization problems. Consequently, the solutions provided by metaheuristic algorithms for optimization problems are termed quasi-optimal. The pursuit of improved quasi-optimal solutions for optimization problems has spurred the development of numerous metaheuristic algorithms^7^. These algorithms are employed to handle optimization tasks in various sciences such as combined heat and power economic dispatch^8^, solving general systems of nonlinear equations^9^, numerical optimization problems^10^, semi-submersible platform boom^11^, dynamic positioning system (DPS)^12^, auto drum fashioned brake design^13^, search the optimal parameters for a bucket wheel reclaimer (BWR)^14,15^, medica applications^16^, and Feature Subset Selection (FSS)^17,18^.

The main research question is, according to the countless metaheuristic algorithms introduced so far, what is the primary motivation for introducing newer algorithms based on the need? The No Free Lunch (NFL) theorem^19^ provides a definitive explanation to address this question. The NFL theorem states that achieving acceptable performance with a metaheuristic algorithm for a specific set of optimization problems does not guarantee similar performance for other optimization problems. An algorithm that has shown success in solving particular optimization problems may fail when applied to others. The NFL theorem highlights that no single metaheuristic algorithm can claim to be the best optimizer for all optimization problems. The NFL theorem serves as a catalyst for ongoing research in the field of metaheuristic algorithms, inspiring researchers to continually innovate and devise more efficient solutions for optimization problems by developing novel algorithms. According to this, the author of this paper, by motivation from the NFL theorem and based on the simulation of human activity in the process of preschool education, has designed a new metaheuristic algorithm to deal with optimization tasks in science.

Our extensive literature review shows no metaheuristic algorithm inspired by the concept of preschool education has yet been developed. This is even though creating an educational and fostering environment in preschool has typical characteristics of intelligent decision-making and optimizing the process. In this study, we aim to bridge this research gap by introducing a novel metaheuristic algorithm named the Preschool Education Optimization Algorithm (PEOA), which draws inspiration from the concept of preschool education.

As mentioned, to provide an effective search process in the problem-solving space, a metaheuristic algorithm must have a high ability in exploration, exploitation, and balancing between them during the search process. In the design of PEOA, by taking separate phases of updating the position of population members to manage exploitation, exploration, and balancing exploitation and exploration, an effort has been made to achieve a powerful and effective search process in the problem-solving space to achieve suitable solutions for optimization problems.

In the design of PEOA, the exploitation ability to manage local search is modeled based on the simulation of the gradual growth of the preschool teacher's educational influence. In this process, the child gradually develops under the teacher's influence. Modeling this gradual learning process by making small changes in the position of PEOA members in the problem-solving space leads to an increase in the exploitation ability of PEOA to manage the local search in the accurate scanning of the problem-solving space near the discovered solutions and promising areas to find better solutions. According to this, PEOA is expected to be effective in exploitation for local search in the problem-solving space.

In the design of PEOA, the exploration ability to manage the global search is modeled based on the simulation of individual knowledge development guided by the teacher. In this process, based on imitating the teacher, the child tries to learn the lesson taught by the teacher. Modeling this learning process by making extensive changes in the position of PEOA members in the problem-solving space leads to an increase in the exploration ability of PEOA to manage the global search in the comprehensive scan of the problem space to prevent the algorithm from getting stuck in local optima and identifying the region containing the global optimum. According to this, PEOA is expected to effectively explore global search in the problem-solving space.

In PEOA design, the simulation of Individual increase of knowledge and self-awareness positively affects the ability to exploit the algorithm for local search. Modeling this process by making small changes in the position of PEOA members leads to improving the algorithm's exploitability to manage the local search.

On the other hand, in the design of PEOA, to manage exploration and exploitation and establish a balance between them during the search process, priority has been given to exploration in the initial iterations so that by making extensive changes in the position of population members, the problem-solving space can be scanned well and the promising areas be identified. Then, by increasing the iterations of the algorithm, priority has been given to exploitation so that by shrinking the range of changes in the position of the population members in the problem-solving space, the algorithm can achieve more effective solutions for the given problem by accurately scanning promising areas. Therefore, the proposed PEOA approach is expected to perform well in exploration, exploitation, and balancing during the search process in the problem-solving space to achieve suitable solutions for optimization problems by managing an effective search process.

The aspects of innovation and novelty of this paper are in the introduction and design of a new human-based metaheuristic algorithm named the Preschool Education Optimization Algorithm (PEOA), which draws its inspiration from the preschool education process. The key contributions of this research are outlined as follows:

- The development of PEOA is grounded in the concept of preschool education.
- PEOA is mathematically modeled through three distinct phases: (i) the gradual growth of the preschool teacher's educational influence, (ii) individual knowledge development guided by the teacher, and (iii) individual increase of knowledge and self-awareness.
- The efficacy of PEOA in solving optimization problems is assessed using fifty-two standard benchmark functions encompassing unimodal, high-dimensional multimodal, and fixed-dimensional multimodal types, as well as the CEC 2017 test suite.
- A comprehensive comparative analysis is carried out to assess the performance of PEOA with ten widely recognized algorithms.
- The practical applicability of PEOA is demonstrated by applying it to twenty-two optimization problems from the CEC 2011 test suite and four engineering design problems, showcasing its effectiveness in real-world scenarios.

The subsequent sections of the paper are thoughtfully structured to present the literature review in the “Literature Review” section, followed by the theoretical framework and mathematical model of the proposed optimizer in the dedicated “Preschool Education Optimization Algorithm” section. The “Simulation Studies and Results” section provides a concise summary of the simulation studies conducted and the corresponding outcomes. The implementation of PEOA in solving real-world applications is presented the “PEOA for real-world applications” section. Conclusions and several proposals for further research are provided the “Conclusion and future works” section.

## Literature review

Metaheuristic algorithms have been developed by taking inspiration from a variety of sources, such as natural phenomena, animal behaviors, biological sciences, physical laws, human interactions, and game rules. These algorithms can be categorized into five main groups based on their fundamental design principles: swarm-based, evolutionary-based, physics-based, human-based, and game-based approaches. Each category represents a distinct approach to problem-solving, leveraging different concepts and techniques.

Swarm-based algorithms draw inspiration from the collective behavior of various organisms in nature, including birds, animals, aquatic creatures, insects, and more. Prominent examples of swarm-based approaches extensively employed for solving optimization problems include Ant Colony Optimization (ACO)^20^, Particle Swarm Optimization (PSO)^21^, Artificial Bee Colony (ABC)^22^, and Firefly Algorithm (FA)^23^. PSO is designed based on the swarming movement observed in flocks of fish and birds as they search for food sources in their environment. ACO leverages the ability of ants to find the optimal route between a food source and their nest. The foraging activities of honey bee colonies inspired the design of ABC. The flashing light behavior exhibited by fireflies, which serves to attract mates and prey through bioluminescence, forms the basis of FA's strategy.

Moreover, the strategies employed by living organisms to locate and obtain food resources, whether through foraging or hunting, have inspired the development of several other swarm-based metaheuristic algorithms. These include the Orca Predation Algorithm (OPA)^24^, Grey Wolf Optimizer (GWO)^25^, Marine Predator Algorithm (MPA)^26^, Tunicate Search Algorithm (TSA)^27^, White Shark Optimizer (WSO)^28^, Walrus Optimization Algorithm (WaOA)^29^, Whale Optimization Algorithm (WOA)^30^, Alpine Skiing.

Optimization (ASO)^31^, Reptile Search Algorithm (RSA)^32^, Conscious neighborhood-based Crow Search Algorithm (CCSA)^33^, Quantum-based Avian Navigation optimizer Algorithm (QANA)^34^, and Starling Murmuration Optimizer (SMO)^35^. These algorithms emulate the swarm intelligence exhibited by living organisms in their search for and acquisition of food resources.

Evolutionary-based algorithms are inspired by biological sciences, genetics, concepts of natural selection, and stochastic operators. Genetic Algorithm (GA)^36^ and Differential Evolution (DE)^37^ are among the most well-known evolutionary-based approaches that are inspired by the reproduction process, the concepts of Darwin's theory of evolution, and the evolutionary operators of selection, crossover, and mutation.

Physics-based algorithms are inspired by the phenomena, laws, forces, processes, and concepts of physics. Simulated Annealing (SA)^38^ is one of the most widely used physics-based methods, whose design is imitated from the annealing process of metals in metallurgy. Several optimization algorithms have been constructed based on motivation in force interaction, concretely on gravitational, electromagnetic, electrostatic, elastic, interatomic, and nuclear forces. Among them belong mainly the Gravitational Search Algorithm (GSA)^39^, Space Gravitational Algorithm (SGA)^40^, Gradient-based Gravitational Search (GGS)^41^, Big Crunch Algorithm (BCA)^42^, Electromagnetic Field Optimization (EFO) ^43^, Coulomb Firefly Algorithm (CFA)^44^, Spring Search Algorithm (SSA)^45^, Central Force Optimization (CFO)^46^, Atom Search Optimization (ASO)^47^, and Nuclear Reaction Optimization (NRO)^48^.

Some other physics-based metaheuristic algorithms are Water Cycle Algorithm (WCA)^49^, Equilibrium Optimizer (EO)^50^, Lightning Attachment Procedure Optimization (LAPO)^51^, Flow Regime Algorithm (FRA)^52^, and Multi-Verse Optimizer (MVO)^53^.

Human-based algorithms are inspired by the mutual communication and interactions of humans in social and individual life. Tabu Search (TS)^54^ creates a tabu list to keep track of recently explored solutions and prevent revisiting them, promoting diverse exploration. The algorithm iteratively generates neighboring solutions, evaluates their fitness, and updates the tabu list accordingly. By incorporating aspiration criteria, Tabu Search can escape local optima.

Teaching Learning Based Optimization (TLBO)^55^ is a popular human-based approach inspired by the teaching and learning dynamics between teachers and students in a classroom setting.

In Queueing Search (QS)^56^ algorithm, typical occurrences involve customers actively choosing fast-service queues, where individual customer service is primarily impacted by staff or the customer themselves. Additionally, others may influence customers during service when the queue order lacks strict adherence.

Some other human-based metaheuristic algorithms are: Poor and Rich Optimization (PRO)^57^, Human Mental Search (HMS)^58^, Multi-Leader Optimizer (MLO)^59^, Following Optimization Algorithm (FOA)^60^, Teamwork Optimization Algorithm (TOA)^61^, War Strategy Optimization (WSO)^62^, Chef Based Optimization Algorithm (CBOA)^63^, Coronavirus Mask Protection Algorithm (CMPA)^64^, and Mother Optimization Algorithm (MOA)^65^.

## Preschool education optimization algorithm

In this section, the theory of the proposed Preschool Education Optimization Algorithm (PEOA) approach is described, then its mathematical modeling is presented for use in optimization applications.

### Inspiration and main idea of PEOA

Preschool education plays a crucial role in a child's early development and lays the foundation for their future learning journey. Attending nursery school provides young children with numerous benefits that contribute to their overall growth and well-being^66^.

One of the key advantages of preschool education is the enlargement of the opportunity for social interaction. Children at this age are naturally curious and eager to explore their surroundings. Preschool offers a nurturing environment where they can engage with peers and develop essential social skills. Children are exposed to different subjects, ideas, and challenges through various activities and play-based learning. Conversations with teachers and peers help them articulate their thoughts and emotions effectively, boosting their self-confidence and self-expression ability^67,68^.

In the realm of preschool education, the role of a teacher extends beyond mere instruction, encompassing a dynamic interplay of intelligent processes that shape young minds. Preschool teachers must solve intricate interactions and involve adaptive strategies to enable the full complexity of education in fostering holistic development.

A preschool teacher acts as a guiding force, steering children's curiosity and exploration toward constructive paths. Through structured activities and open-ended play, the teacher creates an environment where children can interact intelligently with their peers, stimulating cognitive growth and social adeptness.

The teacher, attuned to the unique needs of each child, facilitates this journey by encouraging self-expression, supporting decision-making, and promoting autonomy. By fostering such intelligent processes, the preschool teacher empowers children to embrace their individuality and develop a strong sense of identity.

Furthermore, the preschool teacher guides children through challenges, triumphs, and fails and mainly nurtures resilience and problem-solving skills. This echoes the notion that intelligent processes are at play, enabling children to overcome obstacles and emerge stronger.

In sum, the role of a preschool teacher extends far beyond conventional instruction. The teacher cultivates an environment where children learn, explore, and grow by orchestrating an intricate symphony of intelligent processes. As the guiding force behind young learners' development, the preschool teacher empowers children to embark on their own lifelong journey of intellectual curiosity, self-discovery, and personal achievement.

Mathematical modeling of these intelligent interactions in preschool education is the fundamental inspiration in PEOA design.

### Mathematical model of PEOA

The proposed PEOA approach is a population-based technique that can provide suitable solutions for optimization problems in a repetition-based process based on the search power of its members. The PEOA population is formed by the members of a community so that the position of each of these members in the search space suggests values ​​for the decision variables of the problem. Each population member is a candidate solution for the problem, which can be represented using a vector from a mathematical point of view. The PEOA population consisting of these vectors can be represented using a matrix according to Eq. (1).

1 $$X\left( t \right) = \left[ {\begin{array}{*{20}c} {\vec{X}_{1} \left( t \right)} \\ \vdots \\ {\vec{X}_{i} \left( t \right)} \\ \vdots \\ {\vec{X}_{N} \left( t \right)} \\ \end{array} } \right]_{N \times m} = \left[ {\begin{array}{*{20}c} {x_{1,1} \left( t \right)} & \cdots & {x_{1,j} \left( t \right)} & \cdots & {x_{1,m} \left( t \right)} \\ \vdots & \ddots & \vdots & {\mathinner{\mkern2mu\raise1pt\hbox{.}\mkern2mu \raise4pt\hbox{.}\mkern2mu\raise7pt\hbox{.}\mkern1mu}} & \vdots \\ {x_{i,1} \left( t \right)} & \cdots & {x_{i,j} \left( t \right)} & \cdots & {x_{i,m} \left( t \right)} \\ \vdots & {\mathinner{\mkern2mu\raise1pt\hbox{.}\mkern2mu \raise4pt\hbox{.}\mkern2mu\raise7pt\hbox{.}\mkern1mu}} & \vdots & \ddots & \vdots \\ {x_{N,1} \left( t \right)} & \cdots & {x_{N,j} \left( t \right)} & \cdots & {x_{N,m} \left( t \right)} \\ \end{array} } \right]_{N \times m} ,$$

where ${\varvec{X}}(t)$ is the PEOA population matrix, ${\overrightarrow{X}}_{i}(t)$ is the $i$th PEOA’s member, ${x}_{i,j}(t)$ is the value of the $j$th variable determined by the $i$th PEOA’s member, $N$ is the number of PEOA population members, $m$ is the number of problem variables, $t\in \left\{\mathrm{1,2},\dots ,T\right\}$ is the iteration counter (i.e., the number of the actual population) and $T$ is the total number of iterations. At the beginning of the algorithm, the initial position of the PEOA population in the search space is generated randomly using Eq. (2).

2 $${x}_{i,j}\left(1\right)=l{b}_{j}+r \cdot \left(u{b}_{j}-l{b}_{j}\right),i=1,\dots ,N,j=1,\dots ,m,$$

where $r$ is a random number from the uniform distribution in the interval $\left[0, 1\right]$, $l{b}_{j}$ and $u{b}_{j}$ are the lower and upper bound of the $j$th problem variable respectively.

Since each PEOA member is a candidate solution for the problem variables, the objective function of the problem can be calculated based on the proposed values of each PEOA member. Therefore, the calculated values for the objective function of the problem can be represented using a vector according to Eq. (3).

3 $$\overrightarrow{F}(t)={\left[\begin{array}{c}{F}_{1}(t)\\ \vdots \\ {F}_{i}(t)\\ \vdots \\ {F}_{N}(t)\end{array}\right]}_{N\times 1}={\left[\begin{array}{c}F({\overrightarrow{X}}_{1}(t))\\ \vdots \\ F({\overrightarrow{X}}_{i}(t))\\ \vdots \\ F({\overrightarrow{X}}_{N}(t))\end{array}\right]}_{N\times 1},$$

where $\overrightarrow{F}(t)$ is the objective function vector and ${F}_{i}(t)$ is the objective function value based on the $i$th PEOA’s member.

Based on the comparison of the calculated values for the objective function, the member that provides the best value for the objective function is known as the best population member ${\overrightarrow{X}}_{best}(t)$. Considering that in each iteration of PEOA, the position of the population members in the search space is updated, new values for the objective function are calculated. Based on the new values evaluated for the objective function, the best member should also be updated in each iteration.

The process of updating the PEOA population in the search space is perform in three phases (i) the gradual growth of the preschool teacher's educational influence, (ii) individual knowledge development guided by the teacher, and (iii) individual increase of knowledge and self-awareness.

### Phase 1: The gradual growth of the preschool teacher's educational influence (exploitation phase)

It is evident that the role of the teacher changes significantly with the child's age and thus depending on the school education level. At the beginning of the educational process, in the nursery, i.e., at the child's age from 0 to 2 years, the teacher has primarily an upbringing role, and the educational one is insignificant. In the age of child from 2 to 5 years, i.e., in kindergarten, the educational role gradually increases over the caregiving role, and in kindergarten, i.e., in the age of children from 5 to 6 years, the level of teaching influence of the preschool teacher is almost at the same level as during the following primary education.

In the design of PEOA, the best member is considered as the preschool teacher. Because in preschool education, the teacher's influence increases with the passage of time^69^. To simulate this phase of PEOA, first, based on the teacher's impact, a new position is calculated for each PEOA member using Eq. (4). Then, if the value of the objective function is improved in the new position, this new position replaces the previous position of the corresponding member according to Eq. (5).

4 $${\overrightarrow{X}}_{i}^{P1}\left(t+1\right)=\left(1-\frac{t}{T}\right) \cdot {\overrightarrow{X}}_{i}\left(t\right)+\frac{t}{T} \cdot \overrightarrow{K}\left(t\right), i=1,\dots ,N,$$

5 $${\overrightarrow{X}}_{i}(t+1)=\left\{\begin{array}{cc}{\overrightarrow{X}}_{i}^{P1}(t+1),& F({\overrightarrow{X}}_{i}^{P1}(t+1))<{F(\overrightarrow{X}}_{i}(t+1)),\\ {\overrightarrow{X}}_{i}\left(t\right),& \mathrm{else},\end{array}\right.i=1,\dots ,N,$$

where ${\overrightarrow{X}}_{i}^{P1}(t+1)$ is the new calculated position for the $i$th PEOA member based on first phase of PEOA, ${x}_{i,j}^{P1}(t+1)$ is its $j$th dimension, $\vec{K}\left( t \right): = \vec{X}_{best} \left( t \right)$ is the preschool teacher (i.e., the kindergarten teacher), $t$ is the iteration counter, $T$ is the total number of iterations.

### Phase 2: Individual knowledge development guided by the teacher (exploration phase)

In this phase of PEOA, population members are updated based on the modeling children's activities, as children try to imitate the work and take on the teacher's experience to be more successful than their classmates. To simulate this phase of the PEOA, first a new position is calculated for each member of the population based on following the preschool teacher using Eq. (6). This process leads to large shifts in the position of population members, which has a positive effect on exploration and global search in different areas of the problem-solving space. According to Eq. (7), the new position calculated for each member of the population is acceptable if it improves the value of the objective function. Equation (7) is a criterion for performing or not performing the process of updating the position of the PEOA member. Hence, Eq. (7) states that the new position is acceptable for a population member if the value of the objective function is improved in the new position, as the movement of population members in the problem-solving space aims to achieve better solutions and prevent the algorithm from moving toward inappropriate solutions.

6 $${\overrightarrow{X}}_{i}^{P2}\left(t+1\right)={\overrightarrow{X}}_{i}\left(t+1\right)+\overrightarrow{\mathrm{rand}}* \left(\overrightarrow{K}\left(t\right)-{\overrightarrow{\mathrm{rand}}}_{2} *{\overrightarrow{X}}_{i}\left(t+1\right)\right),i=1,\dots ,N,j=1,\dots ,m,$$

7 $$\vec{X}_{i} \left( {t + 1} \right) = \left\{ {\begin{array}{*{20}l} {\vec{X}_{i}^{{P2}} (t + 1),F(\vec{X}_{i}^{{P2}} (t + 1))} \hfill & { < F(\vec{X}_{i} (t + 1)),} \hfill \\ {\vec{X}_{i} (t + 1),else,} \hfill & {} \hfill \\ \end{array} } \right.i = 1, \ldots ,N,$$

where ${\overrightarrow{X}}_{i}^{P2}(t+1)$ is the new calculated position for the $i$ th PEOA member based on second phase of PEOA, ${x}_{i,j}^{P2}(t+1)$ is its $j$ th dimension, $\overrightarrow{\mathrm{rand}}$ is a random vector of the dimension $m$ drawn from the uniform distribution in the interval $\left[0, 1\right],$ and ${\overrightarrow{\mathrm{rand}}}_{2}$ is a random vector of the dimension $m$ generated from the uniform distribution in the set $\left\{\mathrm{1,2}\right\}$.

### Phase 3: Individual increase of knowledge and self-awareness (exploitation phase)

In addition to the influence of the kindergarten teacher, each child tries to increase their self-awareness through different ways such as playing games, analyzing the possibilities, expectations, etc. Increasing self-awareness leads to achieving an ideal state of themselves.

In the third phase of PEOA, the population members are updated based on modeling children's efforts to raise self-awareness. To simulate this phase of PEOA, first, a new position is randomly generated near each member of the population using Eq. (8). This process leads to small changes in the position of population members, which plays an influential role in increasing the PEOA local search and exploitation ability in finding possible better solutions around the discovered solutions. According to Eq. (9), the proposed calculated position for each member of the population is acceptable if it improves the value of the objective function.

8 $${\overrightarrow{X}}_{i}^{P3}\left(t+1\right)={\overrightarrow{X}}_{i}\left(t+1\right)+\overrightarrow{\mathrm{rand}}* \left({\overrightarrow{X}}_{i}\left(t+1\right)-{\overrightarrow{X}}_{i}\left(t\right)\right),i=1,\dots ,N,$$

9 $${\overrightarrow{X}}_{i}(t+1)=\left\{\begin{array}{l}{\overrightarrow{X}}_{i}^{P3}(t+1), F({\overrightarrow{X}}_{i}^{P3}(t+1))<{F(\overrightarrow{X}}_{i}(t+1));\\ {\overrightarrow{X}}_{i}(t+1), else,\end{array}\right.$$

where ${\overrightarrow{X}}_{i}^{P3}(t+1)$ is the new calculated position for the $i$th PEOA member based on third phase of PEOA, ${x}_{i,j}^{P3}(t+1)$ is its $j$th dimension and $\overrightarrow{\mathrm{rand}}$ is a random vector of the dimension $m$ drawn from the uniform distribution in the interval $\left[0, 1\right]$.

#### Repetition process, pseudo-code, and flowchart of PEOA

After updating all PEOA members based on the first to third phases, the first iteration of PEOA is completed. After completing each iteration, the best candidate solution for the problem is updated. Then, based on the new values calculated for the position of the population members and their corresponding objective function, the algorithm enters the next iteration. The process of updating PEOA members using Eqs. (4) to (9) continues until the full implementation of PEOA. At the end, the best candidate solution found during the iterations of the algorithm is presented as the solution to the problem. The pseudo-code of different steps of PEOA implementation is presented in Algorithm 1.

**Algorithm 1:**
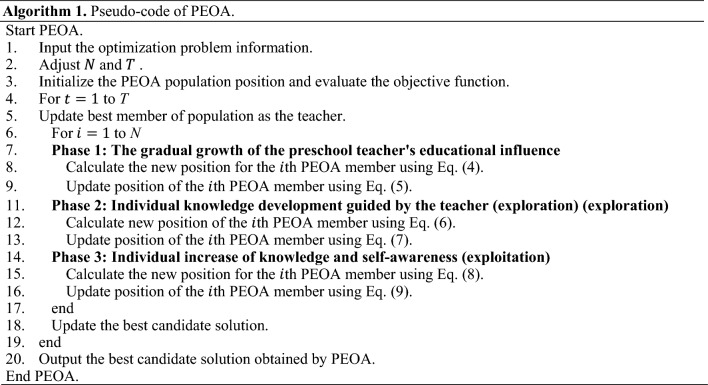
Pseudo-code of PEOA.

### Computational complexity of PEOA

In this subsection, the PEOA computational complexity analysis is discussed. PEOA initialization for an optimization problem has a complexity equal to $O(N \cdot m),$ where $N$ is the number of population members and *m* is the number of decision variables of the problem. In each iteration, PEOA population members are updated in three phases. The PEOA update process has a complexity equal to $O(3N \cdot m \cdot T)$, where $T$ is the total number of iterations of the algorithm. Therefore, the total computational complexity of the proposed PEOA is equal to $O (N \cdot m (3T+1))$.

## Simulation studies and results

In this section, the performance of PEOA in solving optimization problems is evaluated. For this purpose, a set of twenty-three standard benchmark functions of unimodal, high-dimensional multimodal, and fixed-dimensional multimodal types are employed. Full details and explanations of these functions are provided in^70^. In addition, the performance of PEOA in handling the CEC 2017 test suite is also evaluated. Complete information and a detailed description of the CEC 2017 test suite information are available at ^71^. The results of PEOA have been compared with the performance of ten famous algorithms GA, PSO, GSA, TLBO, MVO, GWO, WOA, MPA, TSA, and RSA. The rationale behind selecting these ten metaheuristic algorithms from the plethora of options in the literature can be summarized as follows. The first group, encompassing GA and PSO, are well-known and widely used algorithms. The second group consists of GSA, TLBO, GWO, and MVO, the most cited methods. The third group comprises recently published and widely used methods: WOA, MPA, TSA, and RSA. The values of the control parameters of competing algorithms are provided in Table 1. It should also be mentioned that due to the main advantage of the proposed PEOA approach, which lacks control parameters in its mathematical model, it does not need any parameter tuning process. In addressing the twenty-three standard benchmark functions F1 to F23, the PEOA and competing algorithms are each employed in twenty independent runs where each iteration contains 1000 iterations to optimize each benchmark function. In addressing the CEC 2017 test suite, the proposed PEOA approach and competitor algorithms are employed in fifty-one independent implementations where each execution contains function evaluations (FEs). Simulation results are reported using six indicators: mean, best, worst, standard deviation (std), median, and rank.

**Table 1:** Assigned values to the control parameters of competitor algorithms.

Algorithm	Parameter	Value
RSA	Sensitive parameter	$\beta =0.01$
	Sensitive parameter	$\alpha =0.1$
	Evolutionary Sense $ES$	$ES$: randomly decreasing values between $2$ and $-2$
MPA	Binary vector	$U = 0$ or 1
	Random vector	$R$ is a vector of uniform random numbers in $\left[0, 1\right].$
	Constant number	$P = 0.5$
	Fish Aggregating Devices $FADs$	$FADs = 0.2$
TSA	c1, c2, c3	random numbers from the interval $[0, 1].$
	Pmin	1
	Pmax	4
WOA	$\mathcal{l}$	$\mathcal{l}$ is a random number in $[-1, 1]$
	$r$	$r$ is a random vector in $\left[0, 1\right].$
	Convergence parameter $a$	$a$: Linear reduction from 2 to 0
GWO	Convergence parameter $a$	$a$: Linear reduction from 2 to 0
	wormhole existence probability (WEP)	$\mathrm{min}(\mathrm{WEP}) = 0.2$ and m $\mathrm{ax}(\mathrm{WEP})=1$
MVO	Exploitation accuracy over the iterations $p$	$p = 6$
TLBO	$rand$	$rand$ is a random number from the interval $[0, 1]$
	teaching factor ${T}_{F}$	${T}_{F} = \mathrm{round} \left[(1+rand)\right]$
GSA	Alpha	20
	${G}_{0}$	100
	Rnorm	2
	Rpower	1
PSO	Velocity limit	10% of dimension range
	Topology	Fully connected
	Inertia weight	Linear reduction from 0.9 to 0.1
	Cognitive and social constant	$\left({C}_{1}, {C}_{2}\right)=(2, 2)$
GA	Type	Real coded
	Mutation	Gaussian (Probability = 0.05)
	Crossover	Whole arithmetic ($\mathrm{Probability} = 0.8$,
	Selection	Roulette wheel (Proportionate)

### Qualitative analysis of PEOA

**Figure 1:**
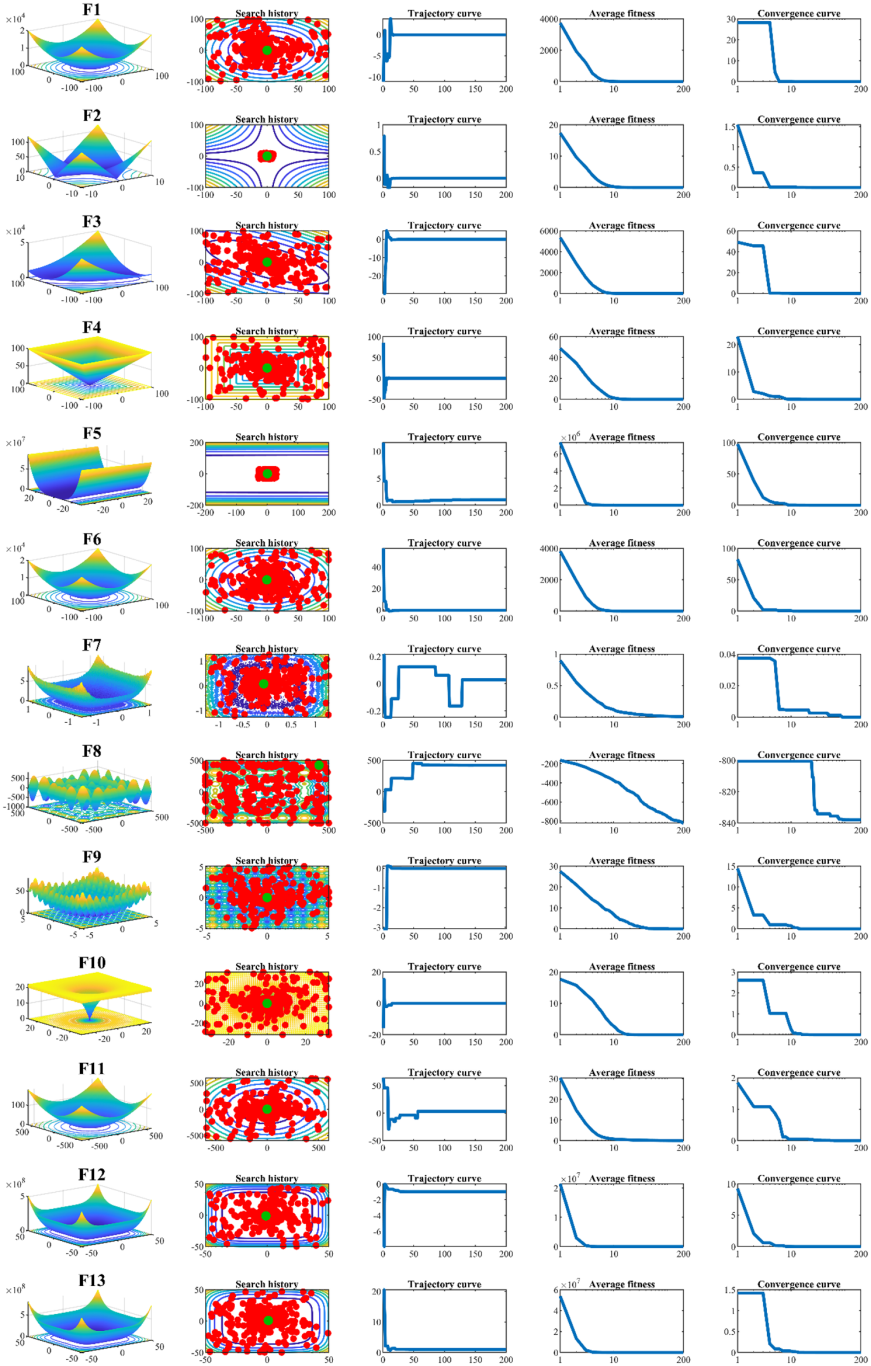
Qualitative analysis of PEOA.

The qualitative analysis results of the proposed PEOA approach in solving some unimodal and multimodal benchmark functions are shown in Fig. 1. In this analysis, four metrics are considered: search history, the trajectory of the first population member in the 1st dimension, the average fitness of the population, and the convergence curve.

The search history metric shows that PEOA searches the problem-solving space well at both global and local levels to discover the original optimal area and converge to the optimal solution. The trajectory metric shows that PEOA creates large changes in the position of the population members in the initial iterations with high exploration ability. Then, with increasing iterations, based on the exploitation ability with minor changes in the position of the population members, it converges towards solutions close to the global optimum. The average fitness metric shows that the population of the algorithm moves towards better solutions during the iterations of the algorithm. The convergence curve metric shows that PEOA has a high convergence speed in solving the problem with a descending trend during the iterations of the algorithm, which indicates the high ability of the proposed algorithm to balance exploration and exploitation.

### Evaluation of unimodal benchmark functions

In order to analyze the exploitation ability of PEOA and competitor algorithms in local search, seven unimodal functions of F1 to F7 are selected. The optimization results of unimodal functions of F1 to F7, using PEOA and competitor algorithms, are reported in Table 2. Based on the obtained results, PEOA with high exploitation ability has converged to the global optimum in solving functions F1, F2, F3, F4, and F6. In solving functions F5 and F7, PEOA is the first best optimizer. Analysis of the simulation results shows that PEOA has a high exploitation ability in local search and compared to competitor algorithms, it has provided superior performance.

**Table 2:** Evaluation results of unimodal functions.

		PEOA	RSA	MPA	TSA	WOA	GWO	MVO	TLBO	GSA	PSO	GA
*F*_1_	Mean	0	0	5.87E−50	2.61E−47	1.69E−152	1.62E−01	1.16E−58	4.15E−74	1.19E−16	0.0362664	34.230951
	Best	0	0.00E+00	2.84E−52	1.24E−50	2.41E−168	9.82E−02	1.12E−60	2.27E−77	6.70E−17	2.073E−05	16.574816
	Worst	0	0	3.48E−49	3.20E−46	2.59E−151	2.27E−01	8.48E−58	7.17E−73	3.64E−16	0.483779	49.532216
	Std	0	0	8.40E−50	7.29E−47	5.84E−152	3.66E−02	2.50E−58	1.59E−73	7.38E−17	0.1078096	10.467117
	Median	0	0	2.42E−50	1.06E−48	1.55E−159	1.58E−01	1.43E−59	3.50E−76	9.51E−17	0.0019868	32.065603
	Rank	1	1	5	6	2	9	4	3	7	8	10
F_2_	Mean	0	0	1.01E−27	9.02E−29	1E−103	2.56E−01	1.07E−34	7.74E−39	4.93E−08	0.9707812	3.3228995
	Best	0	0	5.97E−30	1.72E−30	3.17E−113	1.53E−01	1.76E−35	6.07E−40	3.45E−08	0.1235284	2.2410278
	Worst	0	0	4.31E−27	5.47E−28	1.54E−102	3.73E−01	7.83E−34	4.79E−38	6.48E−08	5.2619108	4.9763673
	Std	0	0	1.13E−27	1.67E−28	3.53E−103	5.40E−02	1.73E−34	1.09E−38	8.64E−09	1.2057648	0.7100882
	Median	0	0	6.94E−28	1.96E−29	5.94E−108	2.53E−01	4.26E−35	4.56E−39	4.98E−08	0.5893712	3.1159037
	Rank	1	1	6	5	2	8	4	3	7	9	10
F_3_	Mean	0	0	1.315E−11	2.48E−10	18,858.845	1.29 E+01	2.649E−14	2.49E−25	5.12 E+02	1054.4577	2195.5586
	Best	0	0	2.652E−16	1.90E−18	2032.3297	4.20 E+00	3.816E−19	2.02E−27	2.11 E+02	27.156302	1279.3432
	Worst	0	0	1.82E−10	4.87E−09	33,406.826	2.07 E+01	4.419E−13	1.29E−24	7.25 E+02	10,037.204	3685.8776
	Std	0	0	4.047E−11	1.09E−09	8589.6075	4.86 E+00	9.822E−14	4.02E−25	1.41 E+02	2392.5678	557.05946
	Median	0	0	5.931E−13	3.76E−14	18,946.43	1.29 E+01	4.75E−16	2.52E−26	5.43 E+02	284.32942	2155.0394
	Rank	1	1	4	5	10	6	3	2	7	8	9
F_4_	Mean	0	0	2.362E−19	1.37E−02	25.164909	5.60E−01	1.229E−14	1.389E−30	1.69 E+00	6.2895249	2.9507205
	Best	0	0	2.66E−20	1.57E−04	0.0159905	2.41E−01	6.529E−16	6.32E−32	1.18E−08	3.0734408	2.0354827
	Worst	0	0	9.464E−19	2.08E−01	66.772408	1.19 E+00	5.365E−14	7.472E−30	6.57 E+00	11.646481	4.3222951
	Std	0	0	2.231E−19	4.59E−02	21.758362	1.99E−01	1.297E−14	1.765E−30	1.67 E+00	2.6353934	0.647295
	Median	0	0	1.621E−19	1.62E−03	23.196445	5.23E−01	7.145E−15	7.971E−31	1.69 E+00	6.0689463	2.9578029
	Rank	1	1	3	5	10	6	4	2	7	9	8
F_5_	Mean	0.0004425	8.6946598	23.549145	28.63252	27.239329	576.06487	26.523222	26.678885	43.865848	85.688829	465.03333
	Best	4.577E−07	1.575E−28	22.951247	27.130444	26.744112	27.656243	25.534928	25.726694	24.573464	9.50 E+00	259.28355
	Worst	0.0032013	28.990103	24.176305	29.081852	28.73628	2095.1885	27.900939	27.97673	312.33572	174.484	836.20012
	Std	0.000918	13.62634	0.3563753	0.4518025	0.554927	747.12125	0.5842817	0.6800463	64.674449	50.026782	174.72182
	Median	5.048E−05	1.299E−26	23.548934	28.822767	27.012342	230.13846	26.214753	26.37307	26.300628	7.51 E+01	405.1663
	Rank	1	2	3	7	6	11	4	5	8	9	10
F_6_	Mean	0	6.6398929	1.43E−09	3.8285078	0.0832617	0.1433226	0.508549	1.142094	1.22E−16	2.7244767	33.438664
	Best	0	4.16 E+00	5.07E−10	2.802989	0.0115735	8.98E−02	1.907E−05	0.4090807	5.64E−17	6.881E−05	17.553177
	Worst	0	7.2500944	3.28E−09	5.0317017	0.2700173	0.2076179	1.250753	2.0796222	2.09E−16	54.106228	65.046009
	Std	0	0.8369605	6.47E−10	0.6222018	0.0794334	0.0313493	0.3354954	0.4035729	4.34E−17	12.094085	14.218234
	Median	0	6.9757851	1.38E−09	3.5684783	0.040989	0.1380778	0.5003995	1.1671963	1.07E−16	0.0027714	28.503061
	Rank	1	10	3	9	4	5	6	7	2	8	11
F_7_	Mean	1.328E−05	9.22E−05	0.0006545	0.0046427	0.0015207	0.0122507	0.0008299	0.0023495	0.0532191	0.1673299	1.03E−02
	Best	6.54E−07	1.03E−05	0.0001747	0.0016636	0.0001157	0.0060462	1.64E−04	0.0004908	0.014482	8.17E−02	4.29E−03
	Worst	5.028E−05	0.0003552	0.0021105	0.0167963	0.0059269	0.0180807	0.001983	0.0051277	0.0968921	0.3189685	1.69E−02
	Std	1.438E−05	8.699E−05	0.0004314	0.0035579	0.0015071	0.0032761	0.0004305	0.0013534	0.0257839	6.52E−02	3.68E−03
	Median	3.606E−06	7.175E−05	0.0005529	0.0037002	0.000804	0.0123418	0.0007959	0.0020168	0.0547296	1.62E−01	9.20E−03
	Rank	1	2	3	7	5	9	4	6	10	11	8
Sum rank		7	18	27	44	39	54	29	28	48	62	66
Mean rank		1	2.5714286	3.8571429	6.2857143	5.5714286	7.7142857	4.1428571	4	6.8571429	8.8571429	9.4285714
Total rank		1	2	3	7	6	9	5	4	8	10	11

### Evaluation of high dimensional multimodal benchmark functions

In order to investigate the exploration capability of PEOA and competitor algorithms in solving problems that have a large number of local optima, six high-dimensional multimodal functions of F8 to F13 have been selected. The implementation results of PEOA and competitor algorithms on functions F8 to F13 are presented in Table 3. The simulation results show that PEOA, with its high exploration capability, has provided the global optimum in solving F9 and F11 functions by discovering the main optimal area in search space. In solving functions F8, F10, F12, and F13, PEOA is the first best optimizer by providing optimal global search. The analysis of the high-dimensional multimodal simulation results shows that PEOA has an acceptable ability in exploration and global search, and compared to competitor algorithms, it has provided superior efficiency in optimizing F8 to F13 functions.

**Table 3:** Evaluation results of high-dimensional multimodal functions.

		PEOA	RSA	MPA	TSA	WOA	GWO	MVO	TLBO	GSA	PSO	GA
*F*_8_	Mean	−12,340.563	−5479.3915	−9.76 E+03	−6.09 E+03	−10,835.776	−8.09 E+03	−6.02 E+03	−5.17 E+03	−2.71 E+03	−6452.3236	−8453.7272
	Best	−12,569.487	−5.66 E+03	−1.04 E+04	−7.73 E+03	−12,569.483	−9.23 E+03	−7.81 E+03	−7.52 E+03	−3.58 E+03	−7836.8201	−9627.5786
	Worst	−9015.5801	−5255.8756	−8.83 E+03	−5.23 E+03	−7160.1437	−6.70 E+03	−3.01 E+03	−4.32 E+03	−2.15 E+03	−5185.7055	−7561.2263
	Std	792.57825	141.02969	4.03 E+02	6.47 E+02	1757.8744	7.09 E+02	9.25 E+02	7.25 E+02	4.85 E+02	761.06762	601.23332
	Median	−12,559.187	−5489.1157	−9.81 E+03	−6.10 E+03	−11,531.955	−8.10 E+03	−6.21 E+03	−5.04 E+03	−2.69 E+03	−6400.0566	−8333.607
	Rank	1	9	3	7	2	5	8	10	11	6	4
*F*_9_	Mean	0	0	0.00 E+00	1.61 E+02	0	1.11 E+02	8.28E−01	0.00 E+00	2.77 E+01	62.148683	54.625233
	Best	0	0	0.00 E+00	8.42 E+01	0	4.09 E+01	0.00 E+00	0.00 E+00	1.39 E+01	21.889242	27.874075
	Worst	0	0	0.00 E+00	2.29 E+02	0	1.61 E+02	1.22 E+01	0.00 E+00	3.88 E+01	95.516848	86.612784
	Std	0	0	0.00 E+00	4.21 E+01	0	2.80 E+01	2.78 E+00	0.00 E+00	7.33 E+00	17.331681	16.779213
	Median	0	0	0.00 E+00	1.67 E+02	0	1.11 E+02	0.00 E+00	0.00 E+00	2.84 E+01	67.171546	54.639675
	Rank	1	1	1	7	1	6	2	1	3	5	4
*F*_10_	Mean	8.882E−16	8.882E−16	4.086E−15	2.21 E+00	3.908E−15	8.18E−01	1.563E−14	4.09E−15	7.66E−09	3.2833888	3.5478982
	Best	8.882E−16	8.882E−16	8.882E−16	7.99E−15	8.882E−16	9.40E−02	1.155E−14	8.88E−16	5.32E−09	1.5021109	2.8115873
	Worst	8.882E−16	8.882E−16	4.441E−15	3.45 E+00	7.994E−15	2.91 E+00	1.865E−14	4.44E−15	9.76E−09	5.1787822	4.3093529
	Std	0	0	1.094E−15	1.33 E+00	2.647E−15	8.53E−01	1.739E−15	1.09E−15	1.07E−09	1.0404378	0.3787695
	Median	8.882E−16	8.882E−16	4.441E−15	2.81 E+00	4.441E−15	1.58E−01	1.51E−14	4.44E−15	7.75E−09	3.402404	3.5164526
	Rank	1	1	3	7	2	6	4	3	5	8	9
*F*_11_	Mean	0	0	0	8.98E−03	0.0096082	3.94E−01	0.0034191	0	7.99 E+00	0.0981329	1.4848734
	Best	0	0	0.00 E+00	0.00 E+00	0	2.84E−01	0	0.00 E+00	3.64 E+00	0.0030975	1.241286
	Worst	0	0	0	2.38E−02	0.1097843	5.12E−01	0.017904	0	1.41 E+01	0.3900938	1.743239
	Std	0	0	0	7.61E−03	0.0299058	6.04E−02	0.006312	0	2.79 E+00	0.0920983	0.1329458
	Median	0	0	0	9.92E−03	0	3.93E−01	0	0	7.87 E+00	0.0737275	1.4632312
	Rank	1	1	1	3	4	6	2	1	8	5	7
*F*_12_	Mean	3.137E−08	1.16244	1.57E−07	8.0074137	0.0167428	0.6392239	0.0329332	0.0751404	0.1702449	1.1511137	0.1626114
	Best	4.736E−10	0.557437	3.842E−08	1.0769903	0.0010716	0.000628	0.0065876	0.03806	2.277E−19	3.54E−05	0.0336148
	Worst	1.695E−07	1.6688946	2.913E−07	16.998566	0.1908087	2.4615261	0.0664481	0.1133911	1.0440811	4.5874462	0.5244877
	Std	4.053E−08	0.3387055	6.877E−08	4.5096676	0.042204	0.7935942	0.0159373	0.0184989	0.2682654	1.2331964	0.125602
	Median	1.432E−08	1.106103	1.461E−07	8.0094012	0.0037323	0.3815718	0.0293849	0.0756851	0.0856922	8.52E−01	0.1211823
	Rank	1	10	2	11	3	8	4	5	7	9	6
*F*_13_	Mean	5.337E−07	0.3927599	2.82E−03	2.8688846	0.2466066	0.036827	0.4665474	1.111075	1.42E−02	5.3795073	2.9235075
	Best	7.965E−11	5.35E−31	1.86E−09	1.6791696	0.0158998	1.42E−02	0.2003169	0.5959632	5.42E−18	0.0286689	1.3610917
	Worst	6.244E−06	2.9	1.31E−02	4.1956798	0.6945697	0.0691117	0.8138707	1.6022415	1.18E−01	17.838499	5.2426649
	Std	1.395E−06	0.9634638	5.06E−03	0.6638534	0.1993274	0.0180155	0.1651567	0.296759	2.96E−02	4.3403151	0.9803997
	Median	7.624E−08	8.367E−31	3.62E−09	2.8262039	0.1936396	0.0320078	0.417461	1.1361137	1.32E−17	4.8528038	2.8760768
	Rank	1	6	2	9	5	4	7	8	3	11	10
Sum rank		6	28	12	44	17	35	27	28	37	44	40
Mean rank		1	4.6666667	2	7.3333333	2.8333333	5.8333333	4.5	4.6666667	6.1666667	7.3333333	6.6666667
Total rank		1	5	2	9	3	6	4	5	7	9	8

### Evaluation of fixed dimensional multimodal benchmark functions

In order to evaluate the ability of PEOA and competitor algorithms, in creating a balance between exploration and exploitation during the search process, ten fixed-dimensional multimodal functions of F14 to F23 have been selected. The results of using PEOA and competitor algorithms in optimizing functions of F14 to F23 are released in Table 4. The simulation results show that PEOA is the first best optimizer in solving functions F14, F15, F18, F22, and F23. In solving functions F16, F17, F19, F20, and F21, PEOA and some competitor algorithms have provided similar results for the "mean" index. However, PEOA has provided better performance in solving these functions by providing better values in the "std" index. The analysis of the results of fixed-dimensional multimodal functions, shows that PEOA has a high ability to balance exploration and exploitation, and by providing better results for these functions, it has superior performance compared to competitor algorithms.

**Table 4:** Evaluation results of fixed-dimensional multimodal functions.

		PEOA	RSA	MPA	TSA	WOA	GWO	MVO	TLBO	GSA	PSO	GA
*F*_14_	Mean	0.9980038	3.4539684	1.0092554	7.8684112	1.8331851	0.9980038	4.324466	1.0972091	2.9987282	3.9731498	0.9980252
	Best	0.9980038	1.9920309	0.9980038	0.9980038	0.9980038	0.9980038	0.9980038	0.9980038	0.9980039	0.9980038	0.9980038
	Worst	0.9980038	12.670506	1.186694	13.618609	10.763181	0.9980038	12.670506	2.9821052	8.8409295	17.374407	0.9981704
	Std	7.204E−17	2.8764897	0.0425402	5.30 E+00	2.27 E+00	5.358E−12	4.381902	0.4436585	2.10 E+00	4.7718472	4.683E−05
	Median	0.9980038	2.9296996	0.9980038	10.763181	0.9980038	0.9980038	2.9821052	0.9980039	2.5033123	1.9920309	0.9980041
	Rank	1	8	4	11	6	2	10	5	7	9	3
*F*_15_	Mean	0.0003075	0.0015433	0.0003076	0.0113933	0.0006059	0.003616	0.0083757	0.0014324	0.0025646	0.0007312	0.0057703
	Best	0.0003075	0.0006645	0.0003075	0.0003077	0.0003097	0.0003081	0.0003075	0.000309	0.0014993	0.0003075	0.0007777
	Worst	0.0003075	0.0047431	0.0003081	0.0566213	0.0014888	0.0203633	0.0203633	0.020364	0.0078279	0.0016554	0.0231219
	Std	2.795E−19	0.0010169	1.693E−07	0.0145179	0.0003651	0.0072219	0.0100442	0.0044653	1.30E−03	0.0005582	7.29E−03
	Median	0.0003075	0.0012893	0.0003075	0.0012242	0.0004979	0.0006627	0.0003079	0.0003192	0.0021802	0.0003075	0.0022826
	Rank	1	6	2	11	3	8	10	5	7	4	9
*F*_16_	Mean	−1.0316285	−1.0295767	−1.0316284	−1.0268839	−1.0316285	−1.0316284	−1.0316284	−1.0316265	−1.0316285	−1.0316285	−1.0316246
	Best	−1.0316285	−1.0316241	−1.0316285	−1.0316284	−1.0316285	−1.0316285	−1.0316285	−1.0316284	−1.0316285	−1.0316285	−1.0316284
	Worst	−1.0316285	−1	−1.0316284	−0.9999983	−1.0316285	−1.0316281	−1.0316284	−1.0316221	−1.0316285	−1.0316285	−1.0315803
	Std	2.28E−16	6.99E−03	6.22E−09	1.16E−02	8.88E−11	9.05E−08	1.09E−08	1.689E−06	1.35E−16	1.139E−16	1.07E−05
	Median	−1.031628	−1.031323	−1.031628	−1.031628	−1.031628	−1.031628	−1.031628	−1.031627	−1.031628	−1.031628	−1.031628
	Rank	1	8	3	9	2	5	4	6	1	1	7
*F*_17_	Mean	0.3978874	0.6524339	0.3978904	0.3979203	0.3978882	0.3978875	0.3978878	0.4020569	0.3978874	0.6008624	0.6846271
	Best	0.3978874	0.3980735	0.3978874	0.3978876	0.3978874	0.3978874	0.3978874	0.3978883	0.3978874	0.3978874	0.3978874
	Worst	0.3978874	5.0401083	0.3979483	0.3980115	0.397894	0.3978878	0.3978896	0.4780146	0.3978874	2.7911841	2.7911856
	Std	0	1.0344736	1.361E−05	3.639E−05	1.69E−06	9.65E−08	5.91E−07	1.79E−02	0.00 E+00	0.5557782	0.7504297
	Median	0.3978874	0.4068639	0.3978874	0.3979015	0.3978875	0.3978874	0.3978876	0.3979799	0.3978874	0.3978874	0.3979251
	Rank	1	9	5	6	4	2	3	7	1	8	10
*F*_18_	Mean	3	5.7443072	3	12.450054	3.0000022	3.0000004	3.0000086	3.0000006	3	3	5.7988278
	Best	3	3.0000001	3	3.0000004	3	3	3	3	3	3	3
	Worst	3	30.673417	3	84.00037	3.0000143	3.000002	3.0000341	3.000003	3	3	31.944856
	Std	9.282E−16	8.45 E+00	6.16E−12	2.01 E+01	3.38E−06	5.23E−07	8.73E−06	6.641E−07	2.92E−15	3.05E−15	8.61 E+00
	Median	3	3.0000624	3	3.0000093	3.0000007	3.0000002	3.0000062	3.0000004	3	3	3.0002555
	Rank	1	9	4	11	7	5	8	6	3	2	10
*F*_19_	Mean	−3.8627821	−3.8139309	−3.862775	−3.8623365	−3.8601261	−3.862782	−3.8624179	−3.861235	−3.8627821	−3.8627821	−3.8621168
	Best	−3.8627821	−3.8602946	−3.8627821	−3.8627735	−3.8627751	−3.8627821	−3.8627821	−3.8627227	−3.8627821	−3.8627821	−3.8627818
	Worst	−3.8627821	−3.6907307	−3.8627274	−3.8548841	−3.8549006	−3.8627816	−3.8571253	−3.8547679	−3.8627821	−3.8627821	−3.8522812
	Std	2.278E−15	4.77E−02	1.33E−05	0.0017545	2.76E−03	1.44E−07	0.0012587	2.78E−03	1.95E−15	1.909E−15	2.34E−03
	Median	−3.8627821	−3.8269795	−3.8627807	−3.8627462	−3.8610067	−3.8627821	−3.8627759	−3.8624013	−3.8627821	−3.8627821	−3.8627486
	Rank	1	9	3	5	8	2	4	7	1	1	6
*F*_20_	Mean	−3.3219952	−2.4524549	−3.2764581	−3.2470228	−3.277447	−3.2623959	−3.2553061	−3.2550375	−3.3219952	−3.2314755	−3.1905623
	Best	−3.3219952	−2.9084273	−3.3219942	−3.321657	−3.3219763	−3.321995	−3.3219937	−3.3123713	−3.3219952	−3.3219952	−3.3214569
	Worst	−3.3219952	−1.3580506	−3.1871382	−3.0385252	−3.1247263	−3.2022028	−3.0838111	−3.1004425	−3.3219952	−3.1376417	−2.9293857
	Std	4.201E−16	0.4481596	6.37E−02	0.077203	0.0699833	0.0611473	0.089609	0.0674298	3.81E−16	0.0638091	1.05E−01
	Median	−3.3219952	−2.6255536	−3.3219751	−3.2025788	−3.3210389	−3.2625323	−3.3219883	−3.3023598	−3.3219952	−3.2031021	−3.1813661
	Rank	1	10	3	7	2	4	5	6	1	8	9
*F*_21_	Mean	−10.1532	−5.0551961	−10.1532	−6.873716	−7.9890962	−7.8805998	−8.6347348	−6.4122769	−7.2467412	−5.3944473	−5.0833003
	Best	−10.1532	−5.0551966	−10.1532	−10.117408	−10.152658	−10.153184	−10.153137	−9.4639595	−10.1532	−10.1532	−9.2069449
	Worst	−10.1532	−5.0551957	−10.1532	−2.6458549	−2.6300523	−2.6304666	−5.0551976	−4.2720878	−2.6828604	−2.6304717	−2.40525
	Std	2.512E−15	2.82E−07	1.911E−15	2.9489362	2.7612154	2.9377491	2.3790359	1.8103759	3.35 E+00	3.33 E+00	2.43 E+00
	Median	−10.1532	−5.0551961	−10.1532	−5.053119	−10.143263	−10.153115	−10.152562	−6.0844842	−10.1532	−3.8918163	−4.7675773
	Rank	1	10	1	6	3	4	2	7	5	8	9
*F*_22_	Mean	−10.402941	−5.087668	−10.137177	−8.3972057	−7.2909308	−8.9620982	−10.402473	−7.574999	−10.402941	−7.6324445	−7.5554948
	Best	−10.402941	−5.0876712	−10.402941	−10.395506	−10.402795	−10.402923	−10.402743	−9.0751508	−10.402941	−10.402941	−10.135923
	Worst	−10.402941	−5.0876667	−5.0876718	−1.8245242	−2.7658537	−2.7658951	−10.401922	−4.3675644	−10.402941	−1.837593	−2.5803561
	Std	3.645E−15	1.118E−06	1.19 E+00	3.049536	2.9406323	2.6050683	0.0002372	1.4695838	2.61E−15	3.55 E+00	2.76 E+00
	Median	−10.402941	−5.0876678	−10.402941	−10.188314	−5.087671	−10.402862	−10.40251	−8.1714763	−10.402941	−10.402941	−8.9790329
	Rank	1	11	4	6	10	5	3	8	2	7	9
*F*_23_	Mean	−10.53641	−5.0665353	−10.266013	−6.589476	−8.5229331	−9.189102	−10.535885	−8.004041	−10.130956	−7.4416614	−7.4142736
	Best	−10.53641	−5.1284795	−10.53641	−10.486731	−10.536228	−10.53639	−10.5363	−9.6123791	−10.53641	−10.53641	−10.373484
	Worst	−10.53641	−3.8897104	−5.1284808	−1.6734109	−2.4217305	−5.1284655	−10.535235	−4.3247641	−2.4273352	−2.4273352	−2.3825916
	Std	2.512E−15	0.2769958	1.21 E+00	3.9421149	3.2295616	2.3941647	0.0002859	1.4547394	1.81 E+00	3.89 E+00	2.72 E+00
	Median	−10.53641	−5.1284727	−10.53641	−7.6335237	−10.527443	−10.536345	−10.535962	−8.558502	−10.53641	−10.53641	−8.3131905
	Rank	1	11	3	10	6	5	2	7	4	8	9
Sum rank		10	91	32	82	51	42	51	64	32	56	81
Mean rank		1	9.1	3.2	8.2	5.1	4.2	5.1	6.4	3.2	5.6	8.1
Total rank		1	9	2	8	4	3	4	6	2	5	7

**Figure 2:**
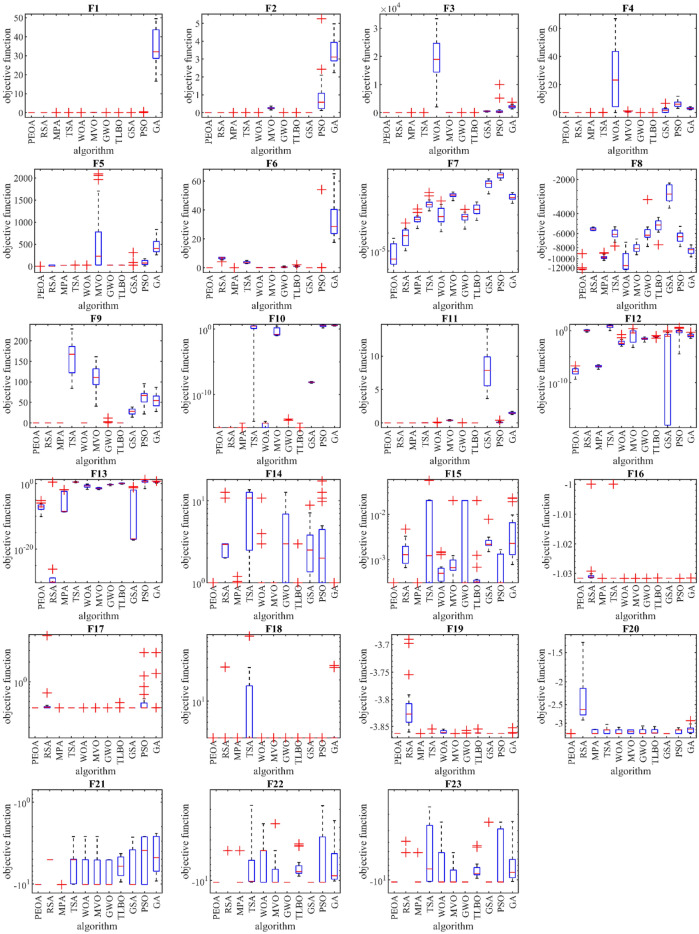
Boxplot of performance of PEOA and competitor algorithms in solving F1 to F23.

Boxplot diagrams resulting from the performance of PEOA and competitor algorithms in optimizing functions F1 to F23 are presented in Fig. 2.

### Evaluation of the CEC 2017 test suite

**Figure 3:**
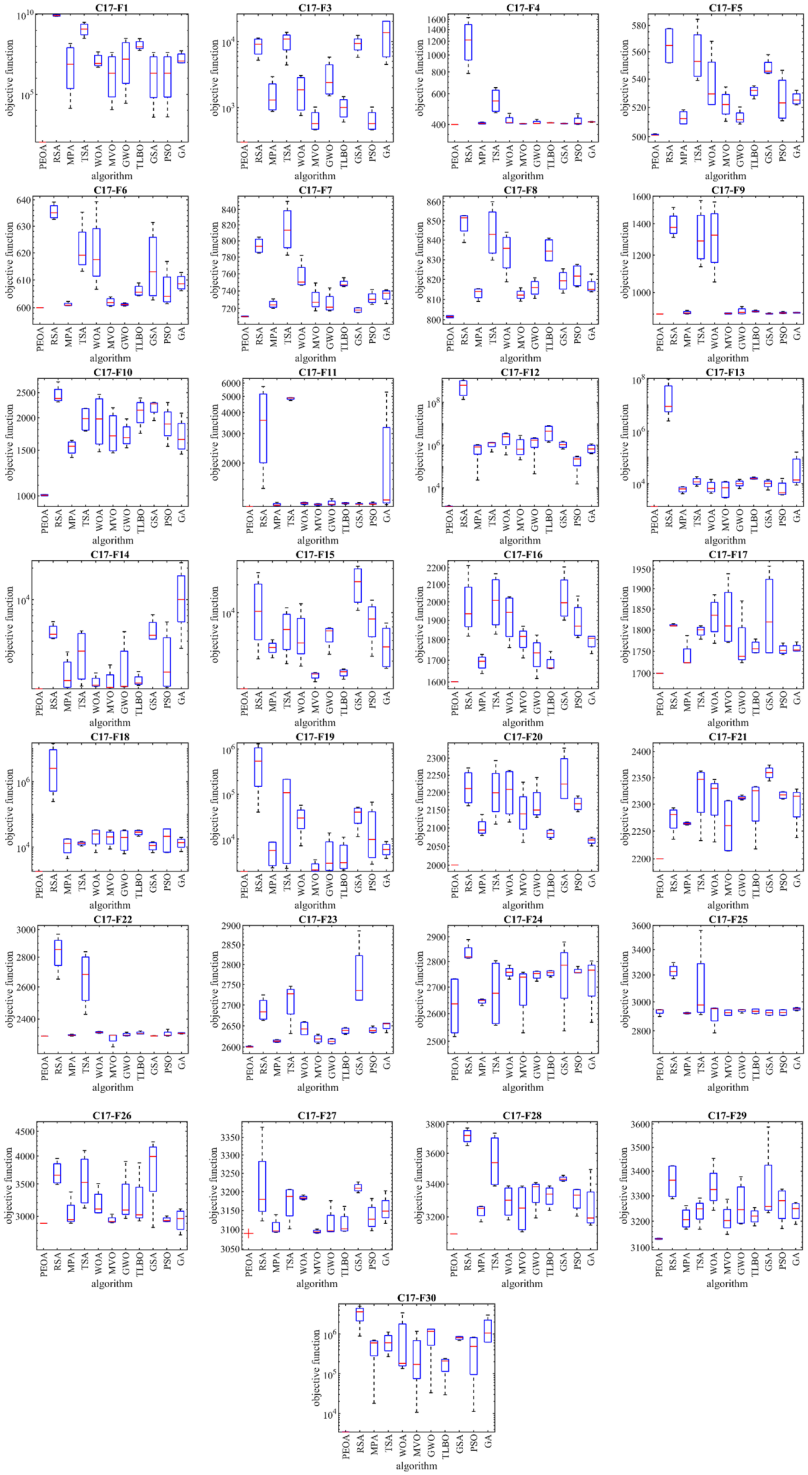
Boxplot of performance of PEOA and competitor algorithms in solving the CEC 2017 test suite.

This subsection evaluates the performance of PEOA and competing algorithms in handling the CEC 2017 test suite. CEC 2017 test suite has thirty standard benchmark objective functions consisting of (i) three unimodal functions of C17-F1 to C17-F3, (ii) seven multimodal functions of C17-F4 to C17-F10, (iii) ten hybrid functions of C17-F11 to C17-F20, and (iv) ten composition functions of C17-F21 to C17-F30. From this test suite, function C17-F2 has been excluded from simulation studies due to its unstable behavior. The results of implementing PEOA and competing algorithms on the CEC 2017 test suite for problem dimensions equal to 10 are reported in Table 5. The boxplot diagrams obtained from the metaheuristic algorithms are drawn in Fig. 3. Based on the optimization results, PEOA is the first best function optimizer: C17-F1, C17-F3 to C17-F21, C17-F23, C17-F24, and C17-F26 to C17-F30. The simulation results show that PEOA has superior performance in handling the CEC 2017 test suite by achieving better results for most of the benchmark functions than competing algorithms.

**Table 5:** Evaluation results of CEC 2017 test suite.

		Peoa	Rsa	Mpa	Tsa	Woa	Mvo	Gwo	Tlbo	Gsa	Pso	Ga
C17-F1	Mean	1.00 e+02	8.75 e+09	4.15 e+07	1.50 e+09	1.68 e+07	1.13 e+07	8.68 e+07	1.37 e+08	1.13 e+07	1.13 e+07	2.14 e+07
	Best	1.00 e+02	7.59 e+09	1.31 e+04	3.19 e+08	4.99 e+06	1.11 e+04	2.73 e+04	5.63 e+07	3.65 e+03	3.86 e+03	9.27 e+06
	Worst	1.00 e+02	1.04 e+10	1.51 e+08	3.25 e+09	4.51 e+07	4.11 e+07	3.15 e+08	3.04 e+08	4.11 e+07	4.11 e+07	5.24 e+07
	Std	0.00 e+00	1.38 e+09	7.90 e+07	1.40 e+09	2.05 e+07	2.15 e+07	1.65 e+08	1.22 e+08	2.15 e+07	2.15 e+07	2.25 e+07
	Median	1.00 e+02	8.49 e+09	7.60 e+06	1.22 e+09	8.60 e+06	2.08 e+06	1.59 e+07	9.44 e+07	2.07 e+06	2.08 e+06	1.20 e+07
	Rank	1	11	7	10	5	4	8	9	2	3	6
C17-F3	Mean	3.00 e+02	8.65 e+03	1.60 e+03	9.98 e+03	1.88 e+03	6.55 e+02	3.02 e+03	1.02 e+03	9.17 e+03	6.55 e+02	1.30 e+04
	Best	3.00 e+02	5.21 e+03	8.77 e+02	4.41 e+03	7.55 e+02	4.57 e+02	1.51 e+03	6.04 e+02	5.75 e+03	4.57 e+02	4.48 e+03
	Worst	3.00 e+02	1.13 e+04	2.93 e+03	1.37 e+04	3.05 e+03	1.02 e+03	5.79 e+03	1.46 e+03	1.23 e+04	1.02 e+03	2.04 e+04
	Std	0.00 e+00	2.99 e+03	1.02 e+03	4.28 e+03	1.22 e+03	2.78 e+02	2.14 e+03	4.07 e+02	2.91 e+03	2.78 e+02	9.04 e+03
	Median	3.00 e+02	9.06 e+03	1.30 e+03	1.09 e+04	1.85 e+03	5.73 e+02	2.39 e+03	1.00 e+03	9.30 e+03	5.73 e+02	1.36 e+04
	Rank	1	8	5	10	6	3	7	4	9	2	11
C17-F4	Mean	4.00 e+02	1.22 e+03	4.07 e+02	5.53 e+02	4.23 e+02	4.04 e+02	4.12 e+02	4.09 e+02	4.05 e+02	4.19 e+02	4.14 e+02
	Best	4.00 e+02	7.84 e+02	4.03 e+02	4.67 e+02	4.08 e+02	4.02 e+02	4.06 e+02	4.09 e+02	4.04 e+02	4.01 e+02	4.12 e+02
	Worst	4.00 e+02	1.64 e+03	4.13 e+02	6.50 e+02	4.64 e+02	4.05 e+02	4.28 e+02	4.11 e+02	4.08 e+02	4.61 e+02	4.17 e+02
	Std	0.00 e+00	3.94 e+02	5.28 e+00	9.75 e+01	2.94 e+01	1.61 e+00	1.18 e+01	1.06 e+00	2.00 e+00	3.06 e+01	2.03 e+00
	Median	4.00 e+02	1.22 e+03	4.06 e+02	5.46 e+02	4.10 e+02	4.05 e+02	4.06 e+02	4.09 e+02	4.05 e+02	4.07 e+02	4.14 e+02
	Rank	1	11	4	10	9	2	6	5	3	8	7
C17-F5	Mean	5.01 e+02	5.65 e+02	5.13 e+02	5.57 e+02	5.37 e+02	5.22 e+02	5.13 e+02	5.31 e+02	5.48 e+02	5.26 e+02	5.26 e+02
	Best	5.01 e+02	5.52 e+02	5.09 e+02	5.39 e+02	5.22 e+02	5.10 e+02	5.08 e+02	5.26 e+02	5.44 e+02	5.11 e+02	5.22 e+02
	Worst	5.02 e+02	5.77 e+02	5.18 e+02	5.85 e+02	5.68 e+02	5.34 e+02	5.20 e+02	5.35 e+02	5.58 e+02	5.46 e+02	5.32 e+02
	Std	5.37e−01	1.59 e+01	5.32 e+00	2.21 e+01	2.34 e+01	1.07 e+01	5.46 e+00	4.30 e+00	6.98 e+00	1.78 e+01	4.88 e+00
	Median	5.01 e+02	5.65 e+02	5.12 e+02	5.53 e+02	5.29 e+02	5.22 e+02	5.12 e+02	5.32 e+02	5.46 e+02	5.23 e+02	5.25 e+02
	Rank	1	11	2	10	8	4	3	7	9	5	6
C17-F6	Mean	6.00 e+02	6.35 e+02	6.01 e+02	6.22 e+02	6.20 e+02	6.02 e+02	6.01 e+02	6.06 e+02	6.15 e+02	6.07 e+02	6.09 e+02
	Best	6.00 e+02	6.33 e+02	6.01 e+02	6.13 e+02	6.07 e+02	6.00 e+02	6.01 e+02	6.04 e+02	6.03 e+02	6.01 e+02	6.06 e+02
	Worst	6.00 e+02	6.39 e+02	6.02 e+02	6.35 e+02	6.39 e+02	6.04 e+02	6.02 e+02	6.09 e+02	6.31 e+02	6.17 e+02	6.13 e+02
	Std	0.00 e+00	3.15 e+00	7.63e−01	1.03 e+01	1.48 e+01	1.66 e+00	5.01e−01	2.29 e+00	1.44 e+01	7.61 e+00	3.15 e+00
	Median	6.00 e+02	6.35 e+02	6.01 e+02	6.19 e+02	6.18 e+02	6.02 e+02	6.01 e+02	6.06 e+02	6.13 e+02	6.04 e+02	6.09 e+02
	Rank	1	11	3	10	9	4	2	5	8	6	7
C17-F7	Mean	7.11 e+02	7.94 e+02	7.25 e+02	8.15 e+02	7.57e+02	7.30e+02	7.26e+02	7.49e+02	7.18e+02	7.32e+02	7.35e+02
	Best	7.11e+02	7.85e+02	7.20e+02	7.82e+02	7.47e+02	7.17e+02	7.17e+02	7.45e+02	7.15e+02	7.25e+02	7.26e+02
	Worst	7.12e+02	8.05e+02	7.31e+02	8.51e+02	7.82e+02	7.49e+02	7.43e+02	7.55e+02	7.21e+02	7.42e+02	7.41e+02
	Std	5.54e−01	1.04e+01	4.99e+00	3.22e+01	1.80e+01	1.46e+01	1.29e+01	4.96e+00	3.09e+00	7.70e+00	7.49e+00
	Median	7.11e+02	7.93e+02	7.24e+02	8.13e+02	7.50e+02	7.27e+02	7.22e+02	7.47e+02	7.18e+02	7.30e+02	7.37e+02
	Rank	1	10	3	11	9	5	4	8	2	6	7
C17-F8	Mean	8.01e+02	8.49e+02	8.13e+02	8.44e+02	8.34e+02	8.12e+02	8.16e+02	8.35e+02	8.19e+02	8.22e+02	8.17e+02
	Best	8.01e+02	8.39e+02	8.09e+02	8.30e+02	8.19e+02	8.09e+02	8.11e+02	8.29e+02	8.13e+02	8.16e+02	8.14e+02
	Worst	8.02e+02	8.53e+02	8.15e+02	8.60e+02	8.44e+02	8.16e+02	8.21e+02	8.41e+02	8.25e+02	8.28e+02	8.23e+02
	Std	6.21e−01	7.19e+00	3.15e+00	1.45e+01	1.18e+01	2.94e+00	4.66e+00	6.77e+00	5.72e+00	6.26e+00	4.42e+00
	Median	8.01e+02	8.52e+02	8.14e+02	8.43e+02	8.36e+02	8.12e+02	8.16e+02	8.34e+02	8.19e+02	8.22e+02	8.15e+02
	Rank	1	11	3	10	8	2	4	9	6	7	5
C17-F9	Mean	9.00e+02	1.39e+03	9.06e+02	1.32e+03	1.31e+03	9.02e+02	9.12e+02	9.12e+02	9.02e+02	9.05e+02	9.06e+02
	Best	9.00e+02	1.31e+03	9.00e+02	1.13e+03	1.05e+03	9.00e+02	9.01e+02	9.09e+02	9.00e+02	9.01e+02	9.04e+02
	Worst	9.00e+02	1.52e+03	9.16e+02	1.57e+03	1.56e+03	9.04e+02	9.33e+02	9.17e+02	9.04e+02	9.11e+02	9.08e+02
	Std	0.00e+00	9.51e+01	7.64e+00	2.03e+02	2.29e+02	2.63e+00	1.65e+01	4.22e+00	2.15e+00	4.46e+00	2.03e+00
	Median	9.00e+02	1.38e+03	9.04e+02	1.29e+03	1.32e+03	9.02e+02	9.07e+02	9.11e+02	9.01e+02	9.05e+02	9.06e+02
	Rank	1	11	6	10	9	3	8	7	2	4	5
C17-F10	Mean	1.01e+03	2.45e+03	1.54e+03	1.98e+03	1.97e+03	1.76e+03	1.72e+03	2.10e+03	2.19e+03	1.91e+03	1.71e+03
	Best	1.00e+03	2.30e+03	1.41e+03	1.78e+03	1.48e+03	1.46e+03	1.53e+03	1.74e+03	1.95e+03	1.55e+03	1.45e+03
	Worst	1.01e+03	2.75e+03	1.64e+03	2.17e+03	2.46e+03	2.19e+03	1.98e+03	2.38e+03	2.29e+03	2.29e+03	2.08e+03
	Std	7.19e+00	2.20e+02	1.08e+02	2.37e+02	5.04e+02	3.66e+02	2.06e+02	2.91e+02	1.75e+02	3.28e+02	2.95e+02
	Median	1.01e+03	2.37e+03	1.55e+03	1.99e+03	1.98e+03	1.70e+03	1.68e+03	2.14e+03	2.26e+03	1.89e+03	1.65e+03
	Rank	1	11	2	8	7	5	4	9	10	6	3
C17-F11	Mean	1.10e+03	3.58e+03	1.13e+03	4.85e+03	1.15e+03	1.13e+03	1.15e+03	1.15e+03	1.14e+03	1.14e+03	2.21e+03
	Best	1.10e+03	1.41e+03	1.11e+03	4.72e+03	1.13e+03	1.11e+03	1.12e+03	1.14e+03	1.13e+03	1.13e+03	1.13e+03
	Worst	1.10e+03	5.72e+03	1.17e+03	4.92e+03	1.17e+03	1.14e+03	1.23e+03	1.16e+03	1.16e+03	1.17e+03	5.30e+03
	Std	0.00e+00	2.10e+03	2.69e+01	9.61e+01	2.02e+01	1.66e+01	5.31e+01	1.29e+01	1.55e+01	2.03e+01	2.23e+03
	Median	1.10e+03	3.60e+03	1.12e+03	4.89e+03	1.15e+03	1.13e+03	1.14e+03	1.15e+03	1.13e+03	1.14e+03	1.20e+03
	Rank	1	10	2	11	7	3	8	6	4	5	9
C17-F12	Mean	1.35e+03	6.08e+08	6.71e+05	1.08e+06	2.21e+06	1.07e+06	1.40e+06	4.53e+06	1.06e+06	1.90e+05	7.04e+05
	Best	1.32e+03	1.35e+08	2.30e+04	4.70e+05	3.44e+05	2.04e+05	4.50e+04	1.36e+06	6.51e+05	1.52e+04	3.94e+05
	Worst	1.44e+03	1.06e+09	1.05e+06	1.34e+06	3.65e+06	2.79e+06	2.19e+06	7.99e+06	1.49e+06	2.98e+05	1.12e+06
	Std	6.19e+01	5.07e+08	4.89e+05	4.42e+05	1.60e+06	1.26e+06	1.02e+06	3.71e+06	3.96e+05	1.33e+05	3.60e+05
	Median	1.33e+03	6.17e+08	8.06e+05	1.25e+06	2.42e+06	6.41e+05	1.68e+06	4.39e+06	1.05e+06	2.23e+05	6.53e+05
	Rank	1	11	3	7	9	6	8	10	5	2	4
C17-F13	Mean	1.31e+03	2.96e+07	6.02e+03	1.23e+04	7.87e+03	7.14e+03	1.02e+04	1.57e+04	1.00e+04	7.04e+03	4.82e+04
	Best	1.30e+03	2.46e+06	4.06e+03	7.89e+03	4.18e+03	2.82e+03	6.46e+03	1.45e+04	5.63e+03	3.64e+03	8.72e+03
	Worst	1.31e+03	9.83e+07	7.59e+03	1.82e+04	1.43e+04	1.20e+04	1.43e+04	1.77e+04	1.41e+04	1.58e+04	1.57e+05
	Std	2.46e+00	4.96e+07	1.71e+03	4.73e+03	4.90e+03	5.27e+03	3.46e+03	1.51e+03	3.78e+03	6.30e+03	7.84e+04
	Median	1.30e+03	8.84e+06	6.22e+03	1.16e+04	6.48e+03	6.85e+03	1.01e+04	1.54e+04	1.02e+04	4.39e+03	1.36e+04
	Rank	1	11	2	8	5	4	7	9	6	3	10
C17-F14	Mean	1.40e+03	4.93e+03	1.99e+03	3.24e+03	1.62e+03	1.67e+03	2.34e+03	1.69e+03	5.11e+03	2.90e+03	1.15e+04
	Best	1.40e+03	4.24e+03	1.44e+03	1.49e+03	1.48e+03	1.43e+03	1.46e+03	1.51e+03	4.17e+03	1.44e+03	3.42e+03
	Worst	1.40e+03	6.15e+03	3.16e+03	5.02e+03	1.99e+03	2.37e+03	4.93e+03	2.05e+03	7.17e+03	6.10e+03	2.25e+04
	Std	5.38e−01	9.45e+02	8.73e+02	1.90e+03	2.63e+02	5.05e+02	1.87e+03	2.66e+02	1.52e+03	2.37e+03	8.67e+03
	Median	1.40e+03	4.66e+03	1.68e+03	3.22e+03	1.52e+03	1.44e+03	1.48e+03	1.59e+03	4.56e+03	2.03e+03	1.00e+04
	Rank	1	9	5	8	2	3	6	4	10	7	11
C17-F15	Mean	1.50e+03	1.27e+04	4.19e+03	6.80e+03	6.13e+03	2.09e+03	5.78e+03	2.24e+03	2.14e+04	8.52e+03	4.69e+03
	Best	1.50e+03	3.16e+03	3.25e+03	2.80e+03	2.64e+03	1.79e+03	3.55e+03	1.91e+03	1.06e+04	3.38e+03	2.50e+03
	Worst	1.50e+03	2.71e+04	5.09e+03	1.13e+04	1.25e+04	2.24e+03	6.85e+03	2.45e+03	3.17e+04	1.36e+04	7.71e+03
	Std	2.54e−01	1.14e+04	8.12e+02	3.95e+03	4.69e+03	2.23e+02	1.64e+03	2.58e+02	1.08e+04	4.60e+03	2.73e+03
	Median	1.50e+03	1.03e+04	4.21e+03	6.55e+03	4.70e+03	2.17e+03	6.35e+03	2.29e+03	2.16e+04	8.54e+03	4.27e+03
	Rank	1	10	4	8	7	2	6	3	11	9	5
C17-F16	Mean	1.60e+03	1.98e+03	1.69e+03	2.00e+03	1.92e+03	1.80e+03	1.73e+03	1.68e+03	2.02e+03	1.90e+03	1.79e+03
	Best	1.60e+03	1.82e+03	1.64e+03	1.83e+03	1.76e+03	1.71e+03	1.62e+03	1.66e+03	1.90e+03	1.81e+03	1.73e+03
	Worst	1.60e+03	2.21e+03	1.73e+03	2.16e+03	2.03e+03	1.87e+03	1.82e+03	1.74e+03	2.20e+03	2.03e+03	1.82e+03
	Std	3.41e−01	1.83e+02	4.08e+01	1.66e+02	1.37e+02	7.15e+01	9.27e+01	4.28e+01	1.46e+02	1.08e+02	4.40e+01
	Median	1.60e+03	1.94e+03	1.70e+03	2.01e+03	1.94e+03	1.82e+03	1.74e+03	1.66e+03	2.00e+03	1.87e+03	1.81e+03
	Rank	1	9	3	10	8	6	4	2	11	7	5
C17-F17	Mean	1.70e+03	1.81e+03	1.74e+03	1.80e+03	1.83e+03	1.83e+03	1.77e+03	1.76e+03	1.84e+03	1.75e+03	1.76e+03
	Best	1.70e+03	1.81e+03	1.72e+03	1.78e+03	1.77e+03	1.77e+03	1.72e+03	1.75e+03	1.75e+03	1.74e+03	1.75e+03
	Worst	1.70e+03	1.81e+03	1.79e+03	1.81e+03	1.89e+03	1.94e+03	1.87e+03	1.78e+03	1.96e+03	1.77e+03	1.77e+03
	Std	1.68e−01	2.87e+00	3.39e+01	1.46e+01	5.29e+01	8.46e+01	7.40e+01	1.64e+01	1.15e+02	1.23e+01	1.08e+01
	Median	1.70e+03	1.81e+03	1.72e+03	1.80e+03	1.84e+03	1.81e+03	1.74e+03	1.76e+03	1.82e+03	1.75e+03	1.75e+03
	Rank	1	8	2	7	9	10	6	5	11	3	4
C17-F18	Mean	1.81e+03	4.90e+06	1.21e+04	1.30e+04	2.26e+04	2.06e+04	1.97e+04	2.80e+04	1.09e+04	2.14e+04	1.36e+04
	Best	1.80e+03	2.47e+05	4.41e+03	1.08e+04	6.83e+03	8.77e+03	6.27e+03	2.15e+04	6.78e+03	6.82e+03	7.30e+03
	Worst	1.82e+03	1.42e+07	1.81e+04	1.48e+04	3.33e+04	3.29e+04	3.32e+04	3.30e+04	1.45e+04	3.63e+04	1.98e+04
	Std	1.09e+01	7.00e+06	7.06e+03	1.88e+03	1.36e+04	1.13e+04	1.48e+04	5.59e+03	3.71e+03	1.80e+04	5.60e+03
	Median	1.80e+03	2.57e+06	1.29e+04	1.31e+04	2.52e+04	2.04e+04	1.97e+04	2.87e+04	1.12e+04	2.12e+04	1.37e+04
	Rank	1	11	3	4	9	7	6	10	2	8	5
C17-F19	Mean	1.90e+03	6.06e+05	5.53e+03	1.09e+05	3.06e+04	2.36e+03	5.34e+03	4.75e+03	3.55e+04	2.22e+04	6.03e+03
	Best	1.90e+03	3.99e+04	2.28e+03	2.22e+03	7.09e+03	1.92e+03	1.94e+03	2.03e+03	1.13e+04	2.76e+03	3.70e+03
	Worst	1.90e+03	1.30e+06	8.64e+03	2.16e+05	5.66e+04	3.44e+03	1.37e+04	1.10e+04	5.09e+04	6.64e+04	8.77e+03
	Std	8.05e−01	6.15e+05	3.72e+03	1.32e+05	2.20e+04	7.87e+02	6.06e+03	4.58e+03	1.91e+04	3.23e+04	2.30e+03
	Median	1.90e+03	5.42e+05	5.60e+03	1.08e+05	2.94e+04	2.04e+03	2.88e+03	2.98e+03	3.98e+04	9.76e+03	5.83e+03
	Rank	1	11	5	10	8	2	4	3	9	7	6
C17-F20	Mean	2.00e+03	2.21e+03	2.10e+03	2.20e+03	2.20e+03	2.14e+03	2.17e+03	2.08e+03	2.24e+03	2.17e+03	2.07e+03
	Best	2.00e+03	2.16e+03	2.08e+03	2.11e+03	2.12e+03	2.06e+03	2.13e+03	2.07e+03	2.18e+03	2.14e+03	2.05e+03
	Worst	2.00e+03	2.27e+03	2.14e+03	2.29e+03	2.26e+03	2.23e+03	2.24e+03	2.10e+03	2.33e+03	2.19e+03	2.07e+03
	Std	0.00e+00	5.60e+01	2.71e+01	8.21e+01	7.86e+01	7.49e+01	5.54e+01	1.31e+01	7.77e+01	2.14e+01	1.08e+01
	Median	2.00e+03	2.21e+03	2.09e+03	2.20e+03	2.21e+03	2.14e+03	2.15e+03	2.08e+03	2.22e+03	2.17e+03	2.07e+03
	Rank	1	10	4	9	8	5	7	3	11	6	2
C17-F21	Mean	2.20e+03	2.27e+03	2.26e+03	2.32e+03	2.31e+03	2.26e+03	2.31e+03	2.30e+03	2.36e+03	2.32e+03	2.30e+03
	Best	2.20e+03	2.24e+03	2.26e+03	2.23e+03	2.23e+03	2.21e+03	2.31e+03	2.22e+03	2.34e+03	2.31e+03	2.24e+03
	Worst	2.20e+03	2.29e+03	2.27e+03	2.36e+03	2.35e+03	2.31e+03	2.32e+03	2.33e+03	2.37e+03	2.32e+03	2.33e+03
	Std	0.00e+00	2.78e+01	2.49e+00	6.57e+01	5.74e+01	5.70e+01	4.04e+00	5.99e+01	1.37e+01	7.53e+00	4.45e+01
	Median	2.20e+03	2.28e+03	2.26e+03	2.35e+03	2.33e+03	2.26e+03	2.31e+03	2.33e+03	2.36e+03	2.32e+03	2.31e+03
	Rank	1	4	3	10	7	2	8	6	11	9	5
C17-F22	Mean	2.30e+03	2.83e+03	2.31e+03	2.66e+03	2.32e+03	2.29e+03	2.31e+03	2.32e+03	2.30e+03	2.31e+03	2.32e+03
	Best	2.30e+03	2.65e+03	2.30e+03	2.43e+03	2.32e+03	2.24e+03	2.30e+03	2.31e+03	2.30e+03	2.30e+03	2.31e+03
	Worst	2.30e+03	2.97e+03	2.31e+03	2.84e+03	2.33e+03	2.31e+03	2.32e+03	2.33e+03	2.30e+03	2.34e+03	2.32e+03
	Std	1.57e−01	1.43e+02	4.48e+00	1.97e+02	5.11e+00	3.56e+01	1.04e+01	8.84e+00	1.36e+00	1.96e+01	3.48e+00
	Median	2.30e+03	2.85e+03	2.30e+03	2.68e+03	2.32e+03	2.31e+03	2.31e+03	2.31e+03	2.30e+03	2.30e+03	2.32e+03
	Rank	2	11	4	10	9	1	5	8	3	6	7
C17-F23	Mean	2.60e+03	2.69e+03	2.61e+03	2.71e+03	2.64e+03	2.62e+03	2.61e+03	2.64e+03	2.77e+03	2.64e+03	2.65e+03
	Best	2.60e+03	2.66e+03	2.61e+03	2.63e+03	2.63e+03	2.61e+03	2.61e+03	2.63e+03	2.71e+03	2.63e+03	2.63e+03
	Worst	2.60e+03	2.72e+03	2.62e+03	2.75e+03	2.66e+03	2.63e+03	2.62e+03	2.65e+03	2.89e+03	2.65e+03	2.66e+03
	Std	1.43e+00	3.07e+01	3.17e+00	5.58e+01	1.83e+01	1.01e+01	6.98e+00	7.94e+00	8.88e+01	7.82e+00	1.19e+01
	Median	2.60e+03	2.68e+03	2.61e+03	2.73e+03	2.64e+03	2.62e+03	2.61e+03	2.64e+03	2.74e+03	2.64e+03	2.66e+03
	Rank	1	9	3	10	7	4	2	5	11	6	8
C17-F24	Mean	2.63e+03	2.84e+03	2.65e+03	2.68e+03	2.76e+03	2.69e+03	2.75e+03	2.75e+03	2.75e+03	2.76e+03	2.73e+03
	Best	2.52e+03	2.81e+03	2.63e+03	2.56e+03	2.73e+03	2.53e+03	2.72e+03	2.74e+03	2.54e+03	2.76e+03	2.57e+03
	Worst	2.73e+03	2.89e+03	2.65e+03	2.80e+03	2.79e+03	2.76e+03	2.76e+03	2.76e+03	2.88e+03	2.78e+03	2.80e+03
	Std	1.26e+02	3.90e+01	1.19e+01	1.44e+02	2.39e+01	1.17e+02	1.96e+01	1.23e+01	1.58e+02	1.33e+01	1.15e+02
	Median	2.64e+03	2.82e+03	2.65e+03	2.68e+03	2.76e+03	2.74e+03	2.75e+03	2.76e+03	2.79e+03	2.76e+03	2.77e+03
	Rank	1	11	2	3	9	4	7	8	6	10	5
C17-F25	Mean	2.93e+03	3.23e+03	2.92e+03	3.11e+03	2.91e+03	2.92e+03	2.94e+03	2.93e+03	2.92e+03	2.93e+03	2.95e+03
	Best	2.90e+03	3.17e+03	2.91e+03	2.91e+03	2.79e+03	2.90e+03	2.92e+03	2.92e+03	2.91e+03	2.90e+03	2.94e+03
	Worst	2.95e+03	3.30e+03	2.93e+03	3.56e+03	2.96e+03	2.94e+03	2.95e+03	2.95e+03	2.94e+03	2.95e+03	2.96e+03
	Std	2.50e+01	5.57e+01	5.15e+00	3.29e+02	9.03e+01	2.35e+01	1.24e+01	1.99e+01	2.18e+01	2.38e+01	1.03e+01
	Median	2.94e+03	3.23e+03	2.92e+03	2.98e+03	2.95e+03	2.92e+03	2.94e+03	2.93e+03	2.92e+03	2.93e+03	2.95e+03
	Rank	6	11	2	10	1	3	8	7	4	5	9
C17-F26	Mean	2.90e+03	3.69e+03	3.04e+03	3.57e+03	3.19e+03	2.95e+03	3.26e+03	3.21e+03	3.78e+03	2.95e+03	2.94e+03
	Best	2.90e+03	3.49e+03	2.90e+03	3.12e+03	3.05e+03	2.91e+03	2.97e+03	2.93e+03	2.84e+03	2.92e+03	2.74e+03
	Worst	2.90e+03	3.95e+03	3.37e+03	4.11e+03	3.51e+03	3.03e+03	3.90e+03	3.87e+03	4.28e+03	3.00e+03	3.11e+03
	Std	4.01e−13	2.29e+02	2.36e+02	4.91e+02	2.34e+02	6.03e+01	4.63e+02	4.78e+02	6.91e+02	3.99e+01	1.75e+02
	Median	2.90e+03	3.65e+03	2.95e+03	3.52e+03	3.11e+03	2.92e+03	3.09e+03	3.02e+03	3.99e+03	2.94e+03	2.97e+03
	Rank	1	10	5	9	6	3	8	7	11	4	2
C17-F27	Mean	3.09e+03	3.22e+03	3.11e+03	3.17e+03	3.18e+03	3.09e+03	3.12e+03	3.12e+03	3.21e+03	3.13e+03	3.15e+03
	Best	3.09e+03	3.12e+03	3.09e+03	3.10e+03	3.18e+03	3.09e+03	3.09e+03	3.10e+03	3.20e+03	3.10e+03	3.12e+03
	Worst	3.09e+03	3.38e+03	3.14e+03	3.21e+03	3.19e+03	3.10e+03	3.18e+03	3.16e+03	3.23e+03	3.18e+03	3.20e+03
	Std	2.84e−13	1.21e+02	2.39e+01	5.27e+01	5.92e+00	4.88e+00	4.34e+01	3.33e+01	1.31e+01	3.89e+01	3.88e+01
	Median	3.09e+03	3.18e+03	3.10e+03	3.19e+03	3.18e+03	3.09e+03	3.10e+03	3.10e+03	3.21e+03	3.13e+03	3.15e+03
	Rank	1	11	3	8	9	2	5	4	10	6	7
C17-F28	Mean	3.10e+03	3.72e+03	3.23e+03	3.55e+03	3.29e+03	3.25e+03	3.34e+03	3.33e+03	3.43e+03	3.31e+03	3.26e+03
	Best	3.10e+03	3.65e+03	3.17e+03	3.39e+03	3.18e+03	3.11e+03	3.19e+03	3.24e+03	3.42e+03	3.20e+03	3.15e+03
	Worst	3.10e+03	3.77e+03	3.26e+03	3.74e+03	3.39e+03	3.39e+03	3.41e+03	3.39e+03	3.46e+03	3.37e+03	3.49e+03
	Std	0.00e+00	5.55e+01	4.68e+01	1.94e+02	1.07e+02	1.59e+02	1.08e+02	7.11e+01	1.70e+01	8.24e+01	1.72e+02
	Median	3.10e+03	3.72e+03	3.25e+03	3.54e+03	3.30e+03	3.25e+03	3.38e+03	3.34e+03	3.43e+03	3.33e+03	3.19e+03
	Rank	1	11	2	10	5	3	8	7	9	6	4
C17-F29	Mean	3.13e+03	3.36e+03	3.21e+03	3.24e+03	3.34e+03	3.21e+03	3.26e+03	3.22e+03	3.33e+03	3.26e+03	3.24e+03
	Best	3.13e+03	3.29e+03	3.17e+03	3.17e+03	3.24e+03	3.15e+03	3.19e+03	3.18e+03	3.23e+03	3.17e+03	3.19e+03
	Worst	3.13e+03	3.42e+03	3.26e+03	3.29e+03	3.45e+03	3.29e+03	3.38e+03	3.25e+03	3.59e+03	3.33e+03	3.27e+03
	Std	2.68e+00	7.80e+01	4.45e+01	5.50e+01	9.42e+01	6.13e+01	9.66e+01	3.21e+01	1.83e+02	7.66e+01	4.18e+01
	Median	3.13e+03	3.36e+03	3.21e+03	3.25e+03	3.32e+03	3.20e+03	3.24e+03	3.22e+03	3.26e+03	3.28e+03	3.25e+03
	Rank	1	11	3	5	10	2	7	4	9	8	6
C17-F30	Mean	3.42e+03	3.28e+06	4.77e+05	6.48e+05	9.72e+05	3.80e+05	9.24e+05	1.72e+05	7.92e+05	4.53e+05	1.43e+06
	Best	3.39e+03	8.85e+05	1.80e+04	2.68e+05	1.35e+05	1.08e+04	3.32e+04	2.95e+04	6.91e+05	1.12e+04	6.23e+05
	Worst	3.44e+03	4.99e+06	6.97e+05	1.12e+06	3.39e+06	1.17e+06	1.34e+06	2.42e+05	8.63e+05	8.30e+05	2.99e+06
	Std	3.00e+01	1.87e+06	3.37e+05	3.94e+05	1.74e+06	5.73e+05	6.62e+05	1.05e+05	8.75e+04	4.53e+05	1.21e+06
	Median	3.42e+03	3.62e+06	5.95e+05	6.02e+05	1.82e+05	1.73e+05	1.16e+06	2.09e+05	8.08e+05	4.85e+05	1.06e+06
	Rank	1	11	5	6	9	3	8	2	7	4	10
Sum rank		35	295	100	252	214	107	174	176	212	168	181
Mean rank		1.21e+00	1.02e+01	3.45e+00	8.69e+00	7.38e+00	3.69e+00	6.00e+00	6.07e+00	7.31e+00	5.79e+00	6.24e+00
Total rank		1	11	2	10	9	3	5	6	8	4	7

### Statistical analysis

Reporting optimization results using mean, best, worst, standard deviation (std), median, and rank indices provides valuable information about the performance of metaheuristic algorithms. However, even with a very low probability, the superiority of one algorithm over several others may be coincidental. Therefore, in this subsection, a statistical analysis is presented on the performance of PEOA and competing algorithms to show whether the superiority of PEOA from a statistical point of view has a significant difference compared to competing algorithms. For this purpose, the Wilcoxon signed-rank test^72^, a non-parametric statistical test, is used. This test uses an index called "$p$-value" to determine whether there is a significant difference between the average of two data samples.

The results of employing the Wilcoxon signed-rank test on the performance of PEOA and competitor algorithms in optimizing the mentioned benchmark functions are reported in Table 6. Based on the statistical analysis results, in cases where "$p$-value" is calculated to be less than 0.05, PEOA has a statistically significant superiority compared to the corresponding competitor algorithm. The statistical analysis shows that PEOA has a significant superiority in solving unimodal benchmark functions, high-dimensional multimodal, fixed-dimensional multimodal, and the CEC 2017 test suite, from a statistical point of view, compared to competitor algorithms.

**Table 6:** Wilcoxon signed-rank test results.

Compared algorithms	Test function type			
	Unimodal	High-multimodal	Fixed-multimodal	CEC 2017 test suite
PEOA vs. RSA	3.78E−06	2.03E−08	1.44E−34	1.97E−21
PEOA vs. MPA	1.01E−24	9.06E−07	9.24E−22	8.03E−19
PEOA vs. TSA	1.01E−24	2.8E−20	1.44E−34	7.99E−21
PEOA vs. WOA	1.01E−24	1.85E−08	1.44E−34	8.60E−21
PEOA vs. MVO	1.01E−24	1.97E−21	1.44E−34	1.26E−19
PEOA vs. GWO	1.01E−24	7.84E−17	1.44E−34	5.23E−21
PEOA vs. TLBO	1.01E−24	1.54E−14	1.44E−34	3.78E−21
PEOA vs. GSA	1.01E−24	2.11E−16	4.81E−13	3.49E−20
PEOA vs. PSO	1.01E−24	1.97E−21	4.12E−16	5.64E−21
PEOA vs. GA	1.01E−24	1.97E−21	1.44E−34	6.72E−20

## Discussion

Metaheuristic algorithms are from the group of stochastic approaches to solve optimization problems that can provide suitable solutions for optimization problems based on random search in the problem-solving space in an iterative process. To have an effective search process, metaheuristic algorithms must have the appropriate power in exploitation, exploration, and balancing during the search process.

Unimodal functions do not have any local optima except the global optimum. For this reason, they are suitable options for measuring the ability to exploit metaheuristic algorithms to manage the local search in the problem-solving space to achieve solutions close to (even matching) the global optimum. Benchmark functions F1 to F7, C17-F1, and C17-F3 are selected from the unimodal type. Based on the optimization results, PEOA has provided the global optimum for the functions F1 to F4, F6, C17-F1, and C17-F3, with high ability in exploitation and powerful local search. Also, PEOA is the first best optimizer for F5 and F7 functions. These results confirm and guarantee the high exploitation power of PEOA to manage local search in problem-solving space. What is evident from the simulation results is that PEOA, by providing better results for unimodal functions and obtaining the rank of the first best generator, has provided a superior performance in competition with competing algorithms, which indicates the exceptional ability of PEOA in exploitation compared to competing algorithms. In addition, the statistical analysis results obtained from the Wilcoxon signed-rank test confirm that PEOA has a significant statistical superiority in competition with the compared algorithms in dealing with unimodal functions and exploitation to manage local search.

High-dimensional multimodal functions of F8 to F13 and C17-F4 to C17-F10 have several local optima in addition to the global optimum. For this reason, these functions are suitable options for measuring the quality of the exploration ability of metaheuristic algorithms to manage the global search in the problem-solving space to prevent the algorithm from getting stuck in local optima and discovering the region containing the global optima. Based on the optimization results, PEOA, with high ability in exploration for global search management, has provided the global optimum for functions F9, F11, C17-F4, C17-F6, and C17-F9. In addition, PEOA is the first best optimizer for functions F8, F10, F12, F13, and C17-F4 to C17-F10. What is evident from the optimization results is that PEOA has a high ability in exploration to manage the global search to identify the region containing the global optimum in the problem-solving space. Based on the simulation results, PEOA has provided superior performance compared to competing algorithms by achieving better results for high-dimensional multimodal functions and C17-F4 to C17-F10 and getting the rank of the first best optimizer, which shows the superior exploration power of the proposed approach in global search management. In addition, the results of the statistical analysis obtained from the Wilcoxon signed-rank test confirm that this superiority of PEOA is significant from a statistical point of view.

Fixed-dimensional multimodal functions F14–F23 have a number of local optima lesser than high-dimensional multimodal functions. For this reason, they are suitable options for evaluating the power of metaheuristic algorithms in balancing exploration and exploitation. Also, benchmark functions C17-F11 to C17-F30 from the CEC 2017 test suite are complex functions that challenge the power of metaheuristic algorithms to balance exploration and exploitation. What is evident from the optimization results is that PEOA, with a high ability to balance between exploration and exploitation, has identified the region containing the global optimum based on the global search and converged to suitable solutions close to the global optimum based on the local search.

Based on the obtained results, PEOA is the first best optimizer in handling F14–F23, C17-F11 to C17-F21, C17-F23, C17-F24, and C17-F27 to C17-F30 compared to competing algorithms. Based on the simulation results, PEOA has provided superior performance compared to competing algorithms to balance exploration and exploitation by achieving better results for most of the benchmark functions and ranking as the first-best optimizer in total. In addition, the statistical analysis results obtained by employing the Wilcoxon signed-rank test confirm that PEOA has a significant statistical superiority compared to competing algorithms to balance exploration and exploitation to deal with high-dimensional multimodal functions of F14 to F23, and functions of C17-F11 to C17-F30 from CEC 2017 test suite.

The main findings from the analysis of the simulation results are that PEOA has high ability in exploitation to manage the local search based on the optimization results of F1 to F7, C17-F1, and C17-F3 functions, has high ability in exploration to manage the global search based on the optimization results of the functions F8 to F13, and C17-F4 to C17-F10, and has a high ability to balance exploration and exploitation based on the optimization results of F14 to F23, and C17-F11 to C17-F30 functions.

## PEOA for real-world applications

In this section, the effectiveness of PEOA in solving optimization problems in real world applications has been evaluated. For this purpose, the ability of PEOA and competitor algorithms in optimizing twenty-two real-world optimization problems from CEC 2011 test suite and four engineering design problems has been challenged. In addressing the CEC 2011 test suite, the proposed PEOA approach and each of the competitor algorithms is implemented in twenty-five independent implementations where each implementation contains 150,000 FEs. In addressing the four engineering design problems, the proposed PEOA approach and each of the competitor algorithms is implemented in twenty independent implementations where each implementation contains 1000 iterations.

In this section, the effectiveness of PEOA in solving optimization problems in real-world applications has been evaluated. For this purpose, the ability of PEOA and competitor algorithms to optimize twenty-two real-world optimization problems from the CEC 2011 test suite and four engineering design problems has been challenged. In addressing the CEC 2011 test suite, the proposed PEOA approach and each competitor algorithm are implemented in twenty-five independent implementations, each containing 150,000 FEs. In addressing the four engineering design problems, the proposed PEOA approach and each of the competitor algorithms are implemented in twenty independent implementations, where each implementation contains 1000 iterations.

### Evaluation of the CEC 2011 test suite

**Figure 4:**
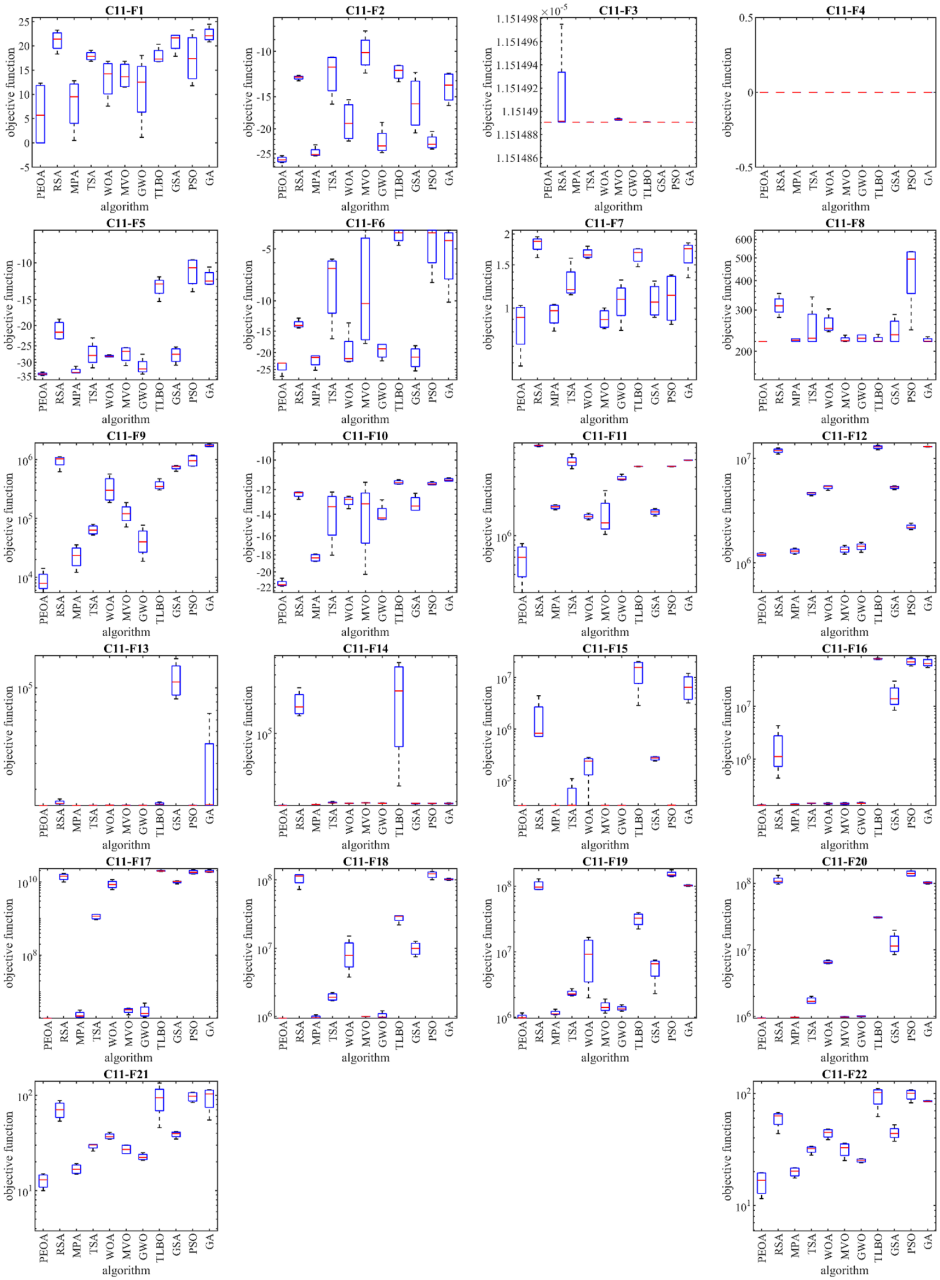
Boxplot of performance of PEOA and competitor algorithms in solving CEC 2011 test suite.

This subsection evaluates the efficiency of PEOA and competing algorithms in handling the CEC 2011 test suite. The CEC 2011 test suite consists of twenty-two constrained optimization problems from real-world applications. Full description, details, and detailed information of the CEC 2011 test suite is available at^73^. The results of employing PEOA and competing algorithms to optimize the CEC 2011 test suite are reported in Table 7. The boxplot diagrams obtained from the performance of the metaheuristic algorithms are plotted in Fig. 4. The optimization results show that PEOA is the first best optimizer for optimization problems C11-F1 to C11-F22. The simulation results show that PEOA had better results than all competitors for all twenty-two optimization problems and was the first-best optimizer to handle the CEC 2011 test suite. In addition, the results obtained from the statistical analysis of the Wilcoxon signed-rank test confirm that PEOA has significant statistical superiority over the competing algorithms to optimize the CEC 2011 test suite.

**Table 7:** Evaluation results of CEC 2011 test suite.

		PEOA	RSA	MPA	TSA	WOA	MVO	GWO	TLBO	GSA	PSO	GA
C11-F1	Mean	5.92E+00	2.11E+01	8.08E+00	1.79E+01	1.32E+01	1.39E+01	1.11E+01	1.79E+01	2.08E+01	1.75E+01	2.24E+01
	Best	2.00E−10	1.83E+01	4.85E−01	1.68E+01	7.56E+00	1.15E+01	1.16E+00	1.68E+01	1.78E+01	1.18E+01	2.08E+01
	Worst	1.23E+01	2.32E+01	1.28E+01	1.91E+01	1.68E+01	1.68E+01	1.80E+01	2.03E+01	2.22E+01	2.33E+01	2.45E+01
	Std	7.40E+00	2.30E+00	5.97E+00	1.04E+00	4.50E+00	2.93E+00	7.71E+00	1.82E+00	2.22E+00	5.61E+00	1.67E+00
	Median	5.69E+00	2.14E+01	9.51E+00	1.78E+01	1.42E+01	1.36E+01	1.25E+01	1.73E+01	2.16E+01	1.74E+01	2.21E+01
	Rank	1	10	2	7	4	5	3	8	9	6	11
C11-F2	Mean	−2.63E+01	−1.26E+01	−2.47E+01	−1.24E+01	−1.89E+01	−1.02E+01	−2.25E+01	−1.20E+01	−1.62E+01	−2.26E+01	−1.39E+01
	Best	−2.71E+01	−1.30E+01	−2.55E+01	−1.60E+01	−2.23E+01	−1.21E+01	−2.47E+01	−1.31E+01	−2.07E+01	−2.39E+01	−1.62E+01
	Worst	−2.54E+01	−1.24E+01	−2.31E+01	−1.05E+01	−1.54E+01	−8.33E+00	−1.89E+01	−1.13E+01	−1.21E+01	−2.05E+01	−1.22E+01
	Std	7.60E−01	2.81E−01	1.20E+00	2.78E+00	3.61E+00	1.71E+00	2.79E+00	9.15E−01	4.20E+00	1.64E+00	2.11E+00
	Median	−2.64E+01	−1.26E+01	−2.52E+01	−1.15E+01	−1.91E+01	−1.01E+01	−2.33E+01	−1.19E+01	−1.60E+01	−2.30E+01	−1.35E+01
	Rank	1	8	2	9	5	11	4	10	6	3	7
C11-F4	Mean	1.15E−05	1.15E−05	1.15E−05	1.15E−05	1.15E−05	1.15E−05	1.15E−05	1.15E−05	1.15E−05	1.15E−05	1.15E−05
	Best	1.15E−05	1.15E−05	1.15E−05	1.15E−05	1.15E−05	1.15E−05	1.15E−05	1.15E−05	1.15E−05	1.15E−05	1.15E−05
	Worst	1.15E−05	1.15E−05	1.15E−05	1.15E−05	1.15E−05	1.15E−05	1.15E−05	1.15E−05	1.15E−05	1.15E−05	1.15E−05
	Std	2.06E−19	4.53E−11	1.63E−15	2.12E−14	5.07E−16	9.04E−13	3.89E−15	7.15E−14	5.07E−16	5.06E−16	5.06E−16
	Median	1.15E−05	1.15E−05	1.15E−05	1.15E−05	1.15E−05	1.15E−05	1.15E−05	1.15E−05	1.15E−05	1.15E−05	1.15E−05
	Rank	1	11	6	8	4	10	7	9	3	2	5
C11-F4	Mean	0.00E+00	0.00E+00	0.00E+00	0.00E+00	0.00E+00	0.00E+00	0.00E+00	0.00E+00	0.00E+00	0.00E+00	0.00E+00
	Best	0.00E+00	0.00E+00	0.00E+00	0.00E+00	0.00E+00	0.00E+00	0.00E+00	0.00E+00	0.00E+00	0.00E+00	0.00E+00
	Worst	0.00E+00	0.00E+00	0.00E+00	0.00E+00	0.00E+00	0.00E+00	0.00E+00	0.00E+00	0.00E+00	0.00E+00	0.00E+00
	Std	0.00E+00	0.00E+00	0.00E+00	0.00E+00	0.00E+00	0.00E+00	0.00E+00	0.00E+00	0.00E+00	0.00E+00	0.00E+00
	Median	0.00E+00	0.00E+00	0.00E+00	0.00E+00	0.00E+00	0.00E+00	0.00E+00	0.00E+00	0.00E+00	0.00E+00	0.00E+00
	Rank	1	1	1	1	1	1	1	1	1	1	1
C11-F5	Mean	−3.41E+01	−2.12E+01	−3.30E+01	−2.76E+01	−2.80E+01	−2.75E+01	−3.15E+01	−1.31E+01	−2.78E+01	−1.11E+01	−1.19E+01
	Best	−3.47E+01	−2.33E+01	−3.37E+01	−3.19E+01	−2.83E+01	−3.11E+01	−3.42E+01	−1.53E+01	−3.10E+01	−1.38E+01	−1.27E+01
	Worst	−3.34E+01	−1.86E+01	−3.13E+01	−2.29E+01	−2.76E+01	−2.55E+01	−2.74E+01	−1.17E+01	−2.53E+01	−9.68E+00	−1.05E+01
	Std	6.07E−01	2.47E+00	1.24E+00	3.97E+00	3.22E−01	2.83E+00	3.12E+00	1.69E+00	2.69E+00	2.08E+00	1.11E+00
	Median	−3.42E+01	−2.15E+01	−3.35E+01	−2.78E+01	−2.81E+01	−2.66E+01	−3.23E+01	−1.26E+01	−2.74E+01	−1.06E+01	−1.22E+01
	Rank	1	8	2	6	4	7	3	9	5	11	10
C11-F6	Mean	−2.41E+01	−1.37E+01	−2.22E+01	−8.83E+00	−1.98E+01	−1.06E+01	−1.96E+01	−4.18E+00	−2.16E+01	−4.95E+00	−5.75E+00
	Best	−2.74E+01	−1.44E+01	−2.53E+01	−1.66E+01	−2.26E+01	−1.77E+01	−2.23E+01	−4.76E+00	−2.55E+01	−7.84E+00	−1.02E+01
	Worst	−2.30E+01	−1.26E+01	−2.09E+01	−5.73E+00	−1.34E+01	−3.89E+00	−1.79E+01	−3.89E+00	−1.82E+01	−3.89E+00	−3.89E+00
	Std	2.39E+00	8.31E−01	2.27E+00	5.63E+00	4.67E+00	7.85E+00	2.29E+00	4.46E−01	3.48E+00	2.09E+00	3.22E+00
	Median	−2.30E+01	−1.39E+01	−2.13E+01	−6.49E+00	−2.17E+01	−1.04E+01	−1.90E+01	−4.03E+00	−2.12E+01	−4.03E+00	−4.47E+00
	Rank	1	6	2	8	4	7	5	11	3	10	9
C11-F7	Mean	8.61E−01	1.82E+00	9.49E−01	1.28E+00	1.67E+00	9.06E−01	1.07E+00	1.64E+00	1.08E+00	1.12E+00	1.66E+00
	Best	5.82E−01	1.61E+00	8.06E−01	1.13E+00	1.59E+00	8.25E−01	8.12E−01	1.47E+00	9.18E−01	8.59E−01	1.33E+00
	Worst	1.03E+00	1.95E+00	1.04E+00	1.60E+00	1.78E+00	1.00E+00	1.30E+00	1.74E+00	1.29E+00	1.36E+00	1.84E+00
	Std	2.17E−01	1.62E−01	1.13E−01	2.32E−01	8.99E−02	9.09E−02	2.18E−01	1.36E−01	1.87E−01	2.86E−01	2.47E−01
	Median	9.18E−01	1.87E+00	9.76E−01	1.19E+00	1.64E+00	8.99E−01	1.09E+00	1.68E+00	1.06E+00	1.13E+00	1.74E+00
	Rank	1	11	3	7	10	2	4	8	5	6	9
C11-F8	Mean	2.20E+02	3.14E+02	2.23E+02	2.54E+02	2.62E+02	2.25E+02	2.27E+02	2.25E+02	2.44E+02	4.42E+02	2.23E+02
	Best	2.20E+02	2.79E+02	2.20E+02	2.20E+02	2.42E+02	2.20E+02	2.20E+02	2.20E+02	2.20E+02	2.47E+02	2.20E+02
	Worst	2.20E+02	3.53E+02	2.26E+02	3.41E+02	3.04E+02	2.34E+02	2.35E+02	2.36E+02	2.87E+02	5.32E+02	2.31E+02
	Std	0.00E+00	3.28E+01	3.92E+00	6.32E+01	3.04E+01	7.17E+00	9.32E+00	8.56E+00	3.44E+01	1.45E+02	5.56E+00
	Median	2.20E+02	3.13E+02	2.23E+02	2.27E+02	2.51E+02	2.22E+02	2.27E+02	2.21E+02	2.35E+02	4.94E+02	2.21E+02
	Rank	1	9	2	7	8	4	5	4	6	10	3
C11-F9	Mean	8.79E+03	9.47E+05	2.34E+04	6.42E+04	3.38E+05	1.24E+05	4.37E+04	3.68E+05	7.35E+05	9.65E+05	1.73E+06
	Best	5.46E+03	6.20E+05	1.21E+04	5.20E+04	1.86E+05	7.13E+04	1.86E+04	3.04E+05	6.30E+05	7.72E+05	1.66E+06
	Worst	1.40E+04	1.11E+06	3.53E+04	7.88E+04	5.67E+05	1.85E+05	7.65E+04	4.70E+05	7.88E+05	1.18E+06	1.83E+06
	Std	4.00E+03	2.40E+05	1.08E+04	1.32E+04	1.87E+05	5.08E+04	2.64E+04	7.82E+04	7.69E+04	2.36E+05	8.95E+04
	Median	7.83E+03	1.03E+06	2.32E+04	6.30E+04	2.99E+05	1.19E+05	3.98E+04	3.48E+05	7.61E+05	9.53E+05	1.71E+06
	Rank	1	9	2	4	6	5	3	7	8	10	11
C11-F10	Mean	−2.15E+01	−1.24E+01	−1.83E+01	−1.42E+01	−1.29E+01	−1.45E+01	−1.40E+01	−1.15E+01	−1.31E+01	−1.16E+01	−1.13E+01
	Best	−2.18E+01	−1.28E+01	−1.87E+01	−1.80E+01	−1.35E+01	−2.03E+01	−1.45E+01	−1.16E+01	−1.36E+01	−1.17E+01	−1.14E+01
	Worst	−2.08E+01	−1.22E+01	−1.79E+01	−1.22E+01	−1.25E+01	−1.15E+01	−1.28E+01	−1.13E+01	−1.23E+01	−1.14E+01	−1.12E+01
	Std	5.13E−01	2.92E−01	4.55E−01	2.83E+00	4.77E−01	4.26E+00	8.62E−01	1.57E−01	7.05E−01	1.21E−01	1.15E−01
	Median	−2.17E+01	−1.22E+01	−1.83E+01	−1.33E+01	−1.28E+01	−1.31E+01	−1.43E+01	−1.15E+01	−1.33E+01	−1.16E+01	−1.13E+01
	Rank	1	8	2	4	7	3	5	10	6	9	11
C11-F11	Mean	5.72E+05	8.34E+06	1.97E+06	5.76E+06	1.57E+06	1.66E+06	3.89E+06	5.11E+06	1.75E+06	5.12E+06	5.92E+06
	Best	2.61E+05	8.13E+06	1.84E+06	4.85E+06	1.45E+06	1.03E+06	3.70E+06	5.08E+06	1.59E+06	5.08E+06	5.88E+06
	Worst	8.29E+05	8.49E+06	2.07E+06	6.85E+06	1.70E+06	2.89E+06	4.26E+06	5.14E+06	1.89E+06	5.16E+06	5.96E+06
	Std	2.68E+05	1.63E+05	1.06E+05	8.89E+05	1.09E+05	9.06E+05	2.73E+05	2.55E+04	1.36E+05	3.51E+04	3.57E+04
	Median	5.99E+05	8.37E+06	1.98E+06	5.67E+06	1.57E+06	1.35E+06	3.80E+06	5.11E+06	1.75E+06	5.12E+06	5.91E+06
	Rank	1	11	5	9	2	3	6	7	4	8	10
C11-F12	Mean	1.20E+06	1.19E+07	1.30E+06	4.62E+06	5.31E+06	1.34E+06	1.43E+06	1.28E+07	5.29E+06	2.22E+06	1.30E+07
	Best	1.16E+06	1.10E+07	1.21E+06	4.40E+06	4.95E+06	1.21E+06	1.26E+06	1.21E+07	5.04E+06	2.08E+06	1.29E+07
	Worst	1.25E+06	1.26E+07	1.38E+06	4.73E+06	5.48E+06	1.47E+06	1.57E+06	1.34E+07	5.45E+06	2.40E+06	1.31E+07
	Std	4.85E+04	7.12E+05	8.16E+04	1.69E+05	2.70E+05	1.15E+05	1.37E+05	5.83E+05	1.95E+05	1.44E+05	1.05E+05
	Median	1.20E+06	1.19E+07	1.30E+06	4.67E+06	5.41E+06	1.34E+06	1.44E+06	1.29E+07	5.34E+06	2.19E+06	1.30E+07
	Rank	1	9	2	6	8	3	4	10	7	5	11
C11-F13	Mean	1.54E+04	1.62E+04	1.55E+04	1.55E+04	1.55E+04	1.55E+04	1.55E+04	1.59E+04	1.15E+05	1.55E+04	2.83E+04
	Best	1.54E+04	1.58E+04	1.55E+04	1.55E+04	1.55E+04	1.55E+04	1.55E+04	1.56E+04	8.39E+04	1.55E+04	1.55E+04
	Worst	1.54E+04	1.71E+04	1.55E+04	1.55E+04	1.56E+04	1.55E+04	1.55E+04	1.64E+04	1.58E+05	1.55E+04	6.67E+04
	Std	9.35E−03	6.66E+02	3.87E+00	1.04E+01	4.51E+01	2.54E+01	9.22E+00	3.76E+02	3.61E+04	2.47E+01	2.76E+04
	Median	1.54E+04	1.59E+04	1.55E+04	1.55E+04	1.55E+04	1.55E+04	1.55E+04	1.58E+04	1.10E+05	1.55E+04	1.56E+04
	Rank	1	9	2	3	7	6	5	8	11	4	10
C11-F14	Mean	1.83E+04	2.05E+05	1.87E+04	1.95E+04	1.92E+04	1.94E+04	1.92E+04	2.77E+05	1.91E+04	1.92E+04	1.91E+04
	Best	1.82E+04	1.52E+05	1.86E+04	1.93E+04	1.91E+04	1.93E+04	1.91E+04	2.90E+04	1.89E+04	1.90E+04	1.89E+04
	Worst	1.84E+04	2.95E+05	1.88E+04	2.00E+04	1.94E+04	1.95E+04	1.94E+04	5.34E+05	1.93E+04	1.93E+04	1.94E+04
	Std	7.36E+01	6.92E+04	8.23E+01	3.72E+02	1.40E+02	9.34E+01	1.61E+02	2.62E+05	2.06E+02	1.31E+02	2.45E+02
	Median	1.83E+04	1.87E+05	1.87E+04	1.94E+04	1.93E+04	1.94E+04	1.92E+04	2.74E+05	1.92E+04	1.92E+04	1.91E+04
	Rank	1	10	2	9	6	8	7	11	3	5	4
C11-F15	Mean	3.29E+04	1.70E+06	3.30E+04	5.21E+04	1.98E+05	3.31E+04	3.31E+04	1.37E+07	2.69E+05	3.33E+04	7.04E+06
	Best	3.28E+04	7.13E+05	3.29E+04	3.31E+04	3.30E+04	3.30E+04	3.30E+04	2.87E+06	2.39E+05	3.33E+04	3.21E+06
	Worst	3.30E+04	4.43E+06	3.30E+04	1.09E+05	2.81E+05	3.32E+04	3.31E+04	2.04E+07	2.90E+05	3.33E+04	1.21E+07
	Std	7.91E+01	1.97E+06	6.26E+01	4.10E+04	1.21E+05	6.06E+01	5.04E+01	8.61E+06	2.59E+04	1.37E+01	4.39E+06
	Median	3.29E+04	8.28E+05	3.30E+04	3.32E+04	2.38E+05	3.31E+04	3.31E+04	1.57E+07	2.74E+05	3.33E+04	6.44E+06
	Rank	1	9	2	6	7	4	3	11	8	5	10
C11-F16	Mean	1.34E+05	1.74E+06	1.39E+05	1.45E+05	1.43E+05	1.43E+05	1.46E+05	7.88E+07	1.66E+07	7.05E+07	6.77E+07
	Best	1.31E+05	4.36E+05	1.37E+05	1.43E+05	1.37E+05	1.35E+05	1.44E+05	7.68E+07	8.44E+06	5.83E+07	5.47E+07
	Worst	1.36E+05	4.31E+06	1.43E+05	1.47E+05	1.48E+05	1.50E+05	1.52E+05	8.11E+07	3.00E+07	8.43E+07	8.66E+07
	Std	2.46E+03	1.88E+06	2.92E+03	2.08E+03	4.91E+03	6.98E+03	4.08E+03	1.94E+06	1.01E+07	1.21E+07	1.46E+07
	Median	1.33E+05	1.12E+06	1.38E+05	1.46E+05	1.43E+05	1.43E+05	1.45E+05	7.87E+07	1.40E+07	6.98E+07	6.48E+07
	Rank	1	7	2	5	4	3	6	11	8	10	9
C11-F17	Mean	1.93E+06	1.38E+10	2.39E+06	1.14E+09	8.59E+09	3.12E+06	3.04E+06	1.98E+10	9.94E+09	1.85E+10	1.94E+10
	Best	1.92E+06	9.88E+09	1.97E+06	9.37E+08	6.13E+09	2.37E+06	2.04E+06	1.90E+10	8.74E+09	1.63E+10	1.81E+10
	Worst	1.94E+06	1.68E+10	3.19E+06	1.30E+09	1.14E+10	3.59E+06	4.94E+06	2.06E+10	1.05E+10	2.13E+10	2.19E+10
	Std	1.23E+04	3.22E+09	5.92E+05	2.01E+08	2.41E+09	5.78E+05	1.41E+06	7.21E+08	8.75E+08	2.46E+09	1.85E+09
	Median	1.92E+06	1.42E+10	2.21E+06	1.15E+09	8.41E+09	3.26E+06	2.59E+06	1.97E+10	1.02E+10	1.81E+10	1.88E+10
	Rank	1	8	2	5	6	4	3	11	7	9	10
C11-F18	Mean	9.42E+05	1.05E+08	9.80E+05	1.94E+06	8.61E+06	9.95E+05	1.03E+06	2.76E+07	9.98E+06	1.20E+08	1.02E+08
	Best	9.38E+05	7.26E+07	9.53E+05	1.70E+06	3.76E+06	9.90E+05	9.67E+05	2.19E+07	7.47E+06	1.00E+08	9.79E+07
	Worst	9.45E+05	1.20E+08	1.05E+06	2.23E+06	1.50E+07	9.97E+05	1.21E+06	2.99E+07	1.26E+07	1.33E+08	1.05E+08
	Std	2.85E+03	2.40E+07	5.35E+04	2.70E+05	5.15E+06	3.14E+03	1.25E+05	4.13E+06	2.47E+06	1.57E+07	3.30E+06
	Median	9.43E+05	1.14E+08	9.57E+05	1.91E+06	7.81E+06	9.96E+05	9.79E+05	2.93E+07	9.94E+06	1.23E+08	1.02E+08
	Rank	1	10	2	5	6	3	4	8	7	11	9
C11-F19	Mean	1.03E+06	1.03E+08	1.17E+06	2.34E+06	9.22E+06	1.47E+06	1.37E+06	3.17E+07	5.72E+06	1.53E+08	1.02E+08
	Best	9.68E+05	8.89E+07	1.09E+06	2.12E+06	1.98E+06	1.16E+06	1.24E+06	2.22E+07	2.30E+06	1.39E+08	9.95E+07
	Worst	1.17E+06	1.29E+08	1.33E+06	2.74E+06	1.66E+07	1.90E+06	1.55E+06	3.95E+07	7.45E+06	1.77E+08	1.05E+08
	Std	1.02E+05	2.04E+07	1.16E+05	2.92E+05	7.43E+06	3.34E+05	1.42E+05	8.08E+06	2.53E+06	1.79E+07	2.49E+06
	Median	9.83E+05	9.67E+07	1.13E+06	2.26E+06	9.16E+06	1.41E+06	1.34E+06	3.25E+07	6.56E+06	1.48E+08	1.02E+08
	Rank	1	10	2	5	7	4	3	8	6	11	9
C11-F20	Mean	9.41E+05	1.11E+08	9.66E+05	1.73E+06	6.57E+06	9.77E+05	1.00E+06	3.08E+07	1.28E+07	1.41E+08	1.02E+08
	Best	9.36E+05	9.72E+07	9.60E+05	1.57E+06	6.20E+06	9.68E+05	9.78E+05	3.01E+07	8.52E+06	1.29E+08	9.74E+07
	Worst	9.47E+05	1.32E+08	9.70E+05	2.00E+06	7.07E+06	9.88E+05	1.02E+06	3.15E+07	1.97E+07	1.53E+08	1.06E+08
	Std	5.16E+03	1.60E+07	4.57E+03	2.21E+05	4.03E+05	1.06E+04	1.78E+04	6.30E+05	5.28E+06	1.46E+07	3.96E+06
	Median	9.41E+05	1.08E+08	9.67E+05	1.68E+06	6.50E+06	9.76E+05	1.00E+06	3.07E+07	1.14E+07	1.41E+08	1.03E+08
	Rank	1	10	2	5	6	3	4	8	7	11	9
C11-F21	Mean	1.27E+01	7.05E+01	1.69E+01	2.92E+01	3.71E+01	2.71E+01	2.26E+01	9.20E+01	3.88E+01	9.65E+01	9.37E+01
	Best	9.97E+00	5.34E+01	1.48E+01	2.60E+01	3.45E+01	2.44E+01	2.08E+01	4.56E+01	3.48E+01	8.38E+01	5.49E+01
	Worst	1.50E+01	8.78E+01	1.92E+01	3.05E+01	4.07E+01	3.01E+01	2.49E+01	1.34E+02	4.14E+01	1.07E+02	1.14E+02
	Std	2.48E+00	1.64E+01	2.19E+00	2.32E+00	2.97E+00	3.34E+00	1.98E+00	3.91E+01	3.18E+00	1.25E+01	2.96E+01
	Median	1.30E+01	7.04E+01	1.67E+01	3.01E+01	3.67E+01	2.70E+01	2.23E+01	9.42E+01	3.95E+01	9.76E+01	1.03E+02
	Rank	1	8	2	5	6	4	3	9	7	11	10
C11-F22	Mean	1.61E+01	5.93E+01	1.99E+01	3.15E+01	4.41E+01	3.16E+01	2.52E+01	9.40E+01	4.44E+01	9.76E+01	8.51E+01
	Best	1.15E+01	4.37E+01	1.75E+01	2.81E+01	3.87E+01	2.51E+01	2.40E+01	6.20E+01	3.74E+01	8.24E+01	8.44E+01
	Worst	1.96E+01	6.76E+01	2.17E+01	3.37E+01	4.81E+01	3.60E+01	2.60E+01	1.11E+02	5.25E+01	1.07E+02	8.64E+01
	Std	4.32E+00	1.15E+01	2.14E+00	2.64E+00	4.61E+00	5.30E+00	1.02E+00	2.36E+01	6.70E+00	1.22E+01	1.02E+00
	Median	1.67E+01	6.29E+01	2.02E+01	3.21E+01	4.48E+01	3.27E+01	2.53E+01	1.02E+02	4.39E+01	1.00E+02	8.48E+01
	Rank	1	8	2	4	6	5	3	10	7	11	9
Sum rank	22	190	51	128	124	105	91	189	134	169	187	
Mean rank	1.00e+00	8.64E+00	2.32E+00	5.82E+00	5.64E+00	4.77E+00	4.14E+00	8.59E+00	6.09E+00	7.68E+00	8.50E+00	
Total rank	1	2	12	4	13	3	11	9	6	7	10	
Wilcoxon: *p*-value		1.37E−15	1.54E−03	4.30E−15	4.62E−15	1.41E−11	1.69E−12	2.94E−15	7.07E−15	1.37E−15	2.01E−15

### Pressure vessel design problem

**Figure 5:**
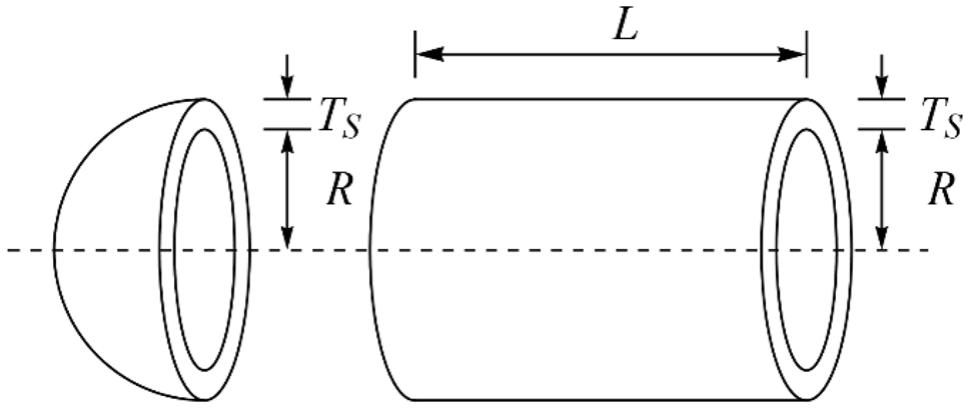
Schematics of the pressure vessel design.

Pressure vessel design is a real-world application where the main goal in this design is to minimize the construction cost. The schematic of this design is presented in Fig. 5. The mathematical model of pressure vessel design is as follows^74^:

$$Consider:X=\left[{x}_{1}, {x}_{2}, {x}_{3}, {x}_{4}\right]=\left[{T}_{s}, {T}_{h}, R, L\right].$$

$$Minimize: f\left(x\right)=0.6224{x}_{1}{x}_{3}{x}_{4}+1.778{x}_{2}{x}_{3}^{2}+3.1661{x}_{1}^{2}{x}_{4}+19.84{x}_{1}^{2}{x}_{3}.$$

$${\begin{array}{c}Subject to:\\ g\end{array}}_{1}\left(x\right)= -{x}_{1}+0.0193{x}_{3} \le 0, {g}_{2}\left(x\right)=-{x}_{2}+0.00954{x}_{3}\le 0,$$

$${g}_{3}\left(x\right)=-\pi {x}_{3}^{2}{x}_{4}-\frac{4}{3}\pi {x}_{3}^{3}+1296000\le 0, {g}_{4}\left(x\right)={x}_{4}-240 \le 0.$$

With

$$0\le {x}_{1},{x}_{2}\le 100 {\mathrm{and} 10\le x}_{3},{x}_{4}\le 200.$$

**Figure 6:**
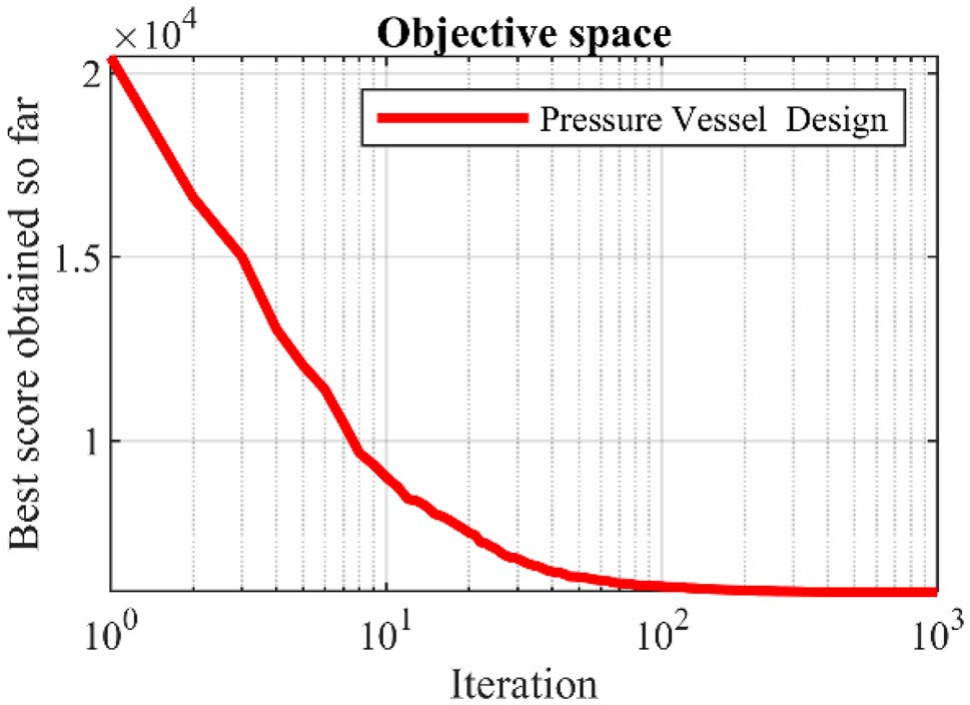
Convergence curves of PEOA on the pressure vessel design.

The results of pressure vessel design optimization are presented in Table 8. Based on the simulation results, PEOA has presented the optimal design of this problem with the values of the design variables equal to (0.778027, 0.384579, 40.31228, 200) and the objective function equal to 5882.9013. The convergence curve of PEOA in the optimization of pressure vessel design is presented in Fig. 6. Analysis of the simulation results shows that PEOA has provided superior performance in pressure vessel design optimization compared to competitor algorithms.

**Table 8:** Evaluation results of the pressure vessel design ($SIs$ are statistical indicators and $DVs$ are design variables).

		PEOA	RSA	MPA	TSA	WOA	GWO	MVO	TLBO	GSA	PSO	GA
$SIs$	Mean	5883.043	12,277.23	5941.73	6495.49	8366.765	6476.968	5906.445	31,739.83	23,227.89	40,801.07	31,420.31
	Best	5882.901	7754.352	5941.73	5925.71	6331.27	5912.494	5888.455	18,534.82	15,456.32	19,543.95	10,111.52
	Worst	5884.245	18,129.78	5941.73	7346.794	11,303.01	7229.541	6034.021	61,688.65	40,575.35	86,266.9	55,069.98
	Std	0.316128	3043.911	8.53E−06	516.9775	1292.405	370.9219	31.59569	11,131.22	6880.321	16,575.37	12,939.3
	Median	5882.903	12,673.37	5941.73	6369.186	8359.384	6416.483	5896.843	28,139.58	21,505.51	37,277.24	29,995.8
	Rank	1	7	3	5	6	4	2	10	8	11	9
$DVs$	*T*_*s*_	0.778027	0.907016	0.778027	0.782743	0.900699	0.791713	0.779624	1.48344	1.474078	1.550955	1.256885
	*T*_*h*_	0.384579	0.673856	0.384579	0.394416	0.429187	0.392455	0.386047	1.891043	1.218961	2.730243	0.734408
	*R*	40.31228	40.65186	40.31228	40.49696	44.44714	41.00272	40.39207	57.20355	72.14816	43.36799	50.50276
	L	200	200	200	197.6681	149.4709	190.6063	198.9133	84.2132	14.55677	168.629	116.7856

### Speed reducer design problem

**Figure 7:**
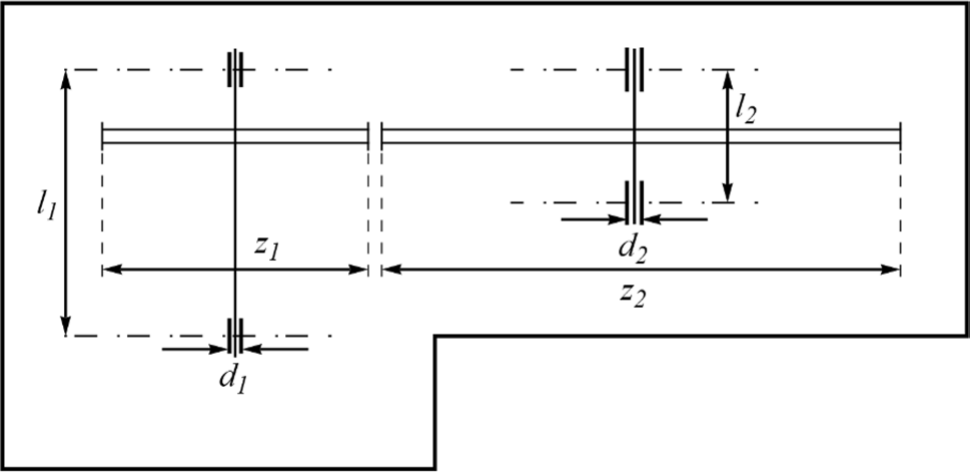
Schematics of the speed reducer design.

Speed reducer design is an engineering challenge whose main goal in this design is to minimize the weight of the speed reducer. The schematic of this design is presented in Fig. 7. The mathematical model of speed reducer design is as follows^75^:

$$Consider:X=\left[{x}_{1,} {x}_{2}, {x}_{3}, {x}_{4}, {x}_{5}{ ,x}_{6} ,{x}_{7}\right]=\left[b, m, p, {l}_{1}, {l}_{2}, {d}_{1}, {d}_{2}\right].$$

$$Minimize:f\left(x\right)=0.7854{x}_{1}{x}_{2}^{2}\left(3.3333{x}_{3}^{2}+14.9334{x}_{3}-43.0934\right)-1.508{x}_{1}\left({x}_{6}^{2}+{x}_{7}^{2}\right)+7.4777\left({x}_{6}^{3}+{x}_{7}^{3}\right)+0.7854\left({x}_{4}{x}_{6}^{2}+{x}_{5}{x}_{7}^{2}\right).$$

$$Subject to:$$

$${g}_{1}\left(x\right)=\frac{27}{{x}_{1}{x}_{2}^{2}{x}_{3}}-1 \le 0, {g}_{2}\left(x\right)=\frac{397.5}{{x}_{1}{x}_{2}^{2}{x}_{3}}-1\le 0,$$

$${g}_{3}\left(x\right)=\frac{1.93{x}_{4}^{3}}{{x}_{2}{x}_{3}{x}_{6}^{4}}-1\le 0, {g}_{4}\left(x\right)=\frac{1.93{x}_{5}^{3}}{{x}_{2}{x}_{3}{x}_{7}^{4}}-1 \le 0,$$

$${g}_{5}\left(x\right)=\frac{1}{110{x}_{6}^{3}}\sqrt{{\left(\frac{745{x}_{4}}{{x}_{2}{x}_{3}}\right)}^{2}+16.9 \cdot {10}^{6}}-1\le 0,$$

$${g}_{6}(x) = \frac{1}{85{x}_{7}^{3}}\sqrt{{\left(\frac{745{x}_{5}}{{x}_{2}{x}_{3}}\right)}^{2}+157.5 \cdot {10}^{6}}-1 \le 0,$$

$${g}_{7}\left(x\right)=\frac{{x}_{2}{x}_{3}}{40}-1 \le 0, {g}_{8}\left(x\right)=\frac{{5x}_{2}}{{x}_{1}}-1 \le 0,$$

$${g}_{9}\left(x\right)=\frac{{x}_{1}}{12{x}_{2}}-1 \le 0, {g}_{10}\left(x\right)=\frac{{1.5x}_{6}+1.9}{{x}_{4}}-1 \le 0,$$

$${g}_{11}\left(x\right)=\frac{{1.1x}_{7}+1.9}{{x}_{5}}-1 \le 0.$$

With

$$2.6\le {x}_{1}\le 3.6, 0.7\le {x}_{2}\le 0.8, 17\le {x}_{3}\le 28, 7.3\le {x}_{4}\le 8.3, 7.8\le {x}_{5}\le 8.3, 2.9\le {x}_{6}\le 3.9, \mathrm{and } 5\le {x}_{7}\le 5.5 .$$

**Figure 8:**
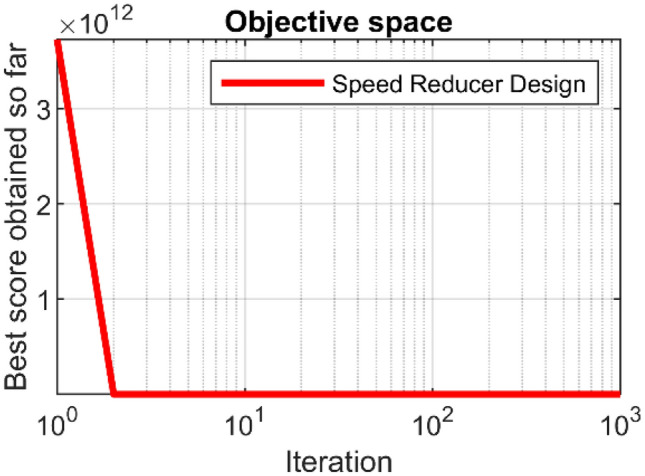
Convergence curves of PEOA on the speed reducer design.

The results of using PEOA and competitor algorithms in optimizing speed reducer design are presented in Table 9. Based on the simulation results, PEOA has presented the optimal design of this problem with the values of the design variables equal to $(3.5, 0.7, 17, 7.3, 7.8, 3.3502147, 5.2866832)$ and the objective function equal to $2996.3482$. The convergence curve of PEOA in speed reducer design optimization is presented in Fig. 8. The comparison of the simulation results indicates the superiority of PEOA against competitor algorithms in order to address the speed reducer design problem.

**Table 9:** Evaluation results of the speed reducer design ($SIs$ are statistical indicators and $DVs$ are design variables).

		PEOA	RSA	MPA	TSA	WOA	GWO	MVO	TLBO	GSA	PSO	GA
SIs	Mean	2996.3482	3265.2077	3026.3117	3036.3735	3245.7174	3034.3775	3005.9781	5.929E+13	3479.3105	1.887E+14	7.652E+13
	Best	2996.3482	3190.4756	3026.3116	3006.6347	3012.0779	3006.1218	2999.5893	3743.3867	3100.1042	5665.6465	3956.2511
	Worst	2996.3482	3363.8734	3026.3117	3059.4124	4508.3061	3091.6465	3015.1587	3.829E+14	3944.4846	1.178E+15	3.215E+14
	Std	3.927E−09	61.523866	6.016E−06	13.543467	412.37918	21.456453	3.7880384	8.617E+13	223.52885	2.737E+14	8.46E+13
	Med	2996.3482	3244.4221	3026.3116	3038.0789	3066.7424	3032.6509	3004.8452	3.181E+13	3407.0976	8.852E+13	5.067E+13
	Rank	1	7	3	5	6	4	2	9	8	11	10
*DVs*	*B*	3.5	3.6	3.5	3.5040015	3.5000578	3.5055119	3.5010175	3.5568875	3.5679922	3.5561928	3.5542606
	*M*	0.7	0.7	0.7	0.7	0.7	0.7	0.7	0.7031734	0.706712	0.7093723	0.7089288
	*P*	17	17	17	17	17	17	17	20.061482	17.012646	27.288704	20.132042
	*L*_1_	7.3	7.3	7.3	7.3	7.6059219	7.3093709	7.4430068	7.5728038	7.5760098	7.8326566	7.8995055
	*L*_2_	7.8	8.3	7.8	8.0976818	7.9384311	7.809156	7.8192616	8.0520916	7.9796192	8.2480258	8.0680724
	*D*_1_	3.3502147	3.3586678	3.3502147	3.3506108	3.3885432	3.378132	3.3529335	3.3494153	3.4574717	3.8570498	3.899926
	*D*_2_	5.2866832	5.5	5.2866832	5.2899388	5.286731	5.2869219	5.2874103	5.4640225	5.3008548	5.420858	5.4554082

### Welded beam design problem

**Figure 9:**
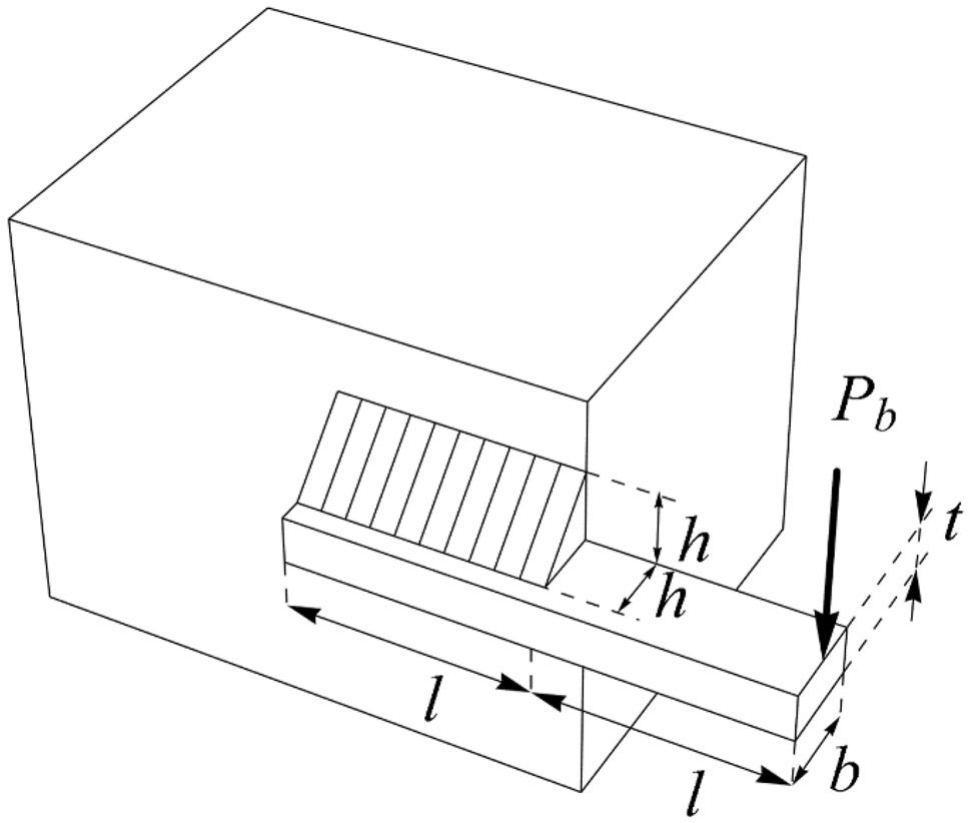
Schematics of the welded beam design.

Welded beam design is a real-world challenge aimed at minimizing fabrication cost. The schematic of this design is presented in Fig. 9. The mathematical model of welded beam design is as follows^23^:

$$Consider: X=\left[{x}_{1}, {x}_{2}, {x}_{3}, {x}_{4}\right]=\left[h, l, t, b\right].$$

$$Minimize:f\left(x\right)=1.10471{x}_{1}^{2}{x}_{2}+0.04811{x}_{3}{x}_{4} \left(14.0+{x}_{2}\right).$$

$$Subject to:$$

$${g}_{1}\left(x\right)= \tau \left(x\right)-13600 \le 0, {g}_{2}\left(x\right)= \sigma \left(x\right)-30000 \le 0,$$

$${g}_{3}\left(x\right)= {x}_{1}-{x}_{4}\le 0, {g}_{4}(x) = 0.10471{x}_{1}^{2}+0.04811{x}_{3}{x}_{4} (14+{x}_{2})-5.0 \le 0,$$

$${g}_{5}\left(x\right)= 0.125 - {x}_{1}\le 0, {g}_{6}\left(x\right)= \delta \left(x\right)- 0.25 \le 0,$$

$${g}_{7}\left(x\right)= 6000 - {p}_{c} \left(x\right)\le 0.$$

where

$$\tau \left(x\right)=\sqrt{{\left({\tau }{\prime}\right)}^{2}+\left(2\tau {\tau }{\prime}\right)\frac{{x}_{2}}{2R}+{\left(\tau "\right)}^{2} }, {\tau }{\prime}=\frac{6000}{\sqrt{2}{x}_{1}{x}_{2}}, \tau "=\frac{MR}{J},$$

$$M=6000\left(14+\frac{{x}_{2}}{2}\right), R=\sqrt{\frac{{x}_{2}^{2}}{4}+{\left(\frac{{x}_{1}+{x}_{3}}{2}\right)}^{2}},$$

$$J=2{x}_{1}{x}_{2}\sqrt{2}\left[\frac{{x}_{2}^{2}}{12}+{\left(\frac{{x}_{1}+{x}_{3}}{2}\right)}^{2}\right] , \sigma \left(x\right)=\frac{504000}{{x}_{4}{x}_{3}^{2}},$$

$$\delta \left(x\right)=\frac{65856000}{\left(30 \cdot 1{0}^{6}\right){x}_{4}{x}_{3}^{3}} , {p}_{c} \left(x\right)=\frac{4.013\left(30 \cdot 1{0}^{6}\right){x}_{3}{x}_{4}^{3}}{1176}\left(1-\frac{{x}_{3}}{112}\right) .$$

With

$$0.1\le {x}_{1}, {x}_{4}\le 2 \mathrm{and }0.1\le {x}_{2}, {x}_{3}\le 10.$$

**Figure 10:**
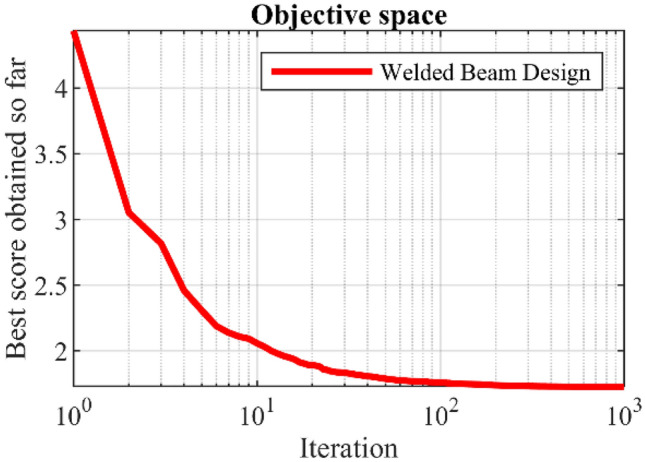
Convergence curves of PEOA on the welded beam design.

The implementation results of PEOA and competitor algorithms in the optimization of welded beam design are presented in Table 10. Based on the simulation results, PEOA has presented the optimal design of this problem with the values of the design variables equal to $(0.20573, 3.470482, 9.036637, 0.20573)$ and the objective function equal to $1.724856$. The convergence curve of PEOA in the optimization of welded beam design is presented in Fig. 10. What is clear from the analysis of simulation results is that compared to competitor algorithms, PEOA has performed better in optimizing welded beam design.

**Table 10:** Evaluation results of the welded beam design ($SIs$ are statistical indicators and $DVs$ are design variables).

		PEOA	RSA	MPA	TSA	WOA	GWO	MVO	TLBO	GSA	PSO	GA
$SIs$	Mean	1.724892	2.341537	1.742101	1.745733	2.301095	1.752466	1.727366	1.95E+13	2.284603	3.6E+14	2.97E+12
	Best	1.724856	1.93442	1.742101	1.730934	1.786288	1.727417	1.72553	3.116494	1.887561	2.562104	2.35562
	Worst	1.724948	3.096801	1.742101	1.765645	4.123886	1.844168	1.729686	2.8E+14	2.67133	6.6E+15	3.78E+13
	Std	3.11E−05	0.262276	9.11E−09	0.007928	0.57756	0.026986	0.001219	6.28E+13	0.23321	1.47E+15	8.82E+12
	Median	1.724884	2.284537	1.742101	1.746652	2.119872	1.743089	1.727121	4.877842	2.279436	6.323903	5.246116
	Rank	1	8	3	4	7	5	2	10	6	11	9
*DVs*	*H*	0.20573	0.160527	0.20573	0.204449	0.201431	0.205378	0.205598	0.469951	0.211075	0.140264	0.222112
	*L*	3.470482	4.41673	3.470489	3.480055	3.842074	3.471103	3.47537	2.567484	3.752937	8.267144	5.01953
	*T*	9.036637	10	9.036624	9.086587	9.169027	9.058422	9.035368	5.712725	8.841494	8.191558	8.659872
	*B*	0.20573	0.204134	0.20573	0.205488	0.205078	0.205633	0.205787	0.546862	0.225499	0.271489	0.262752

### Tension/compression spring design problem

**Figure 11:**
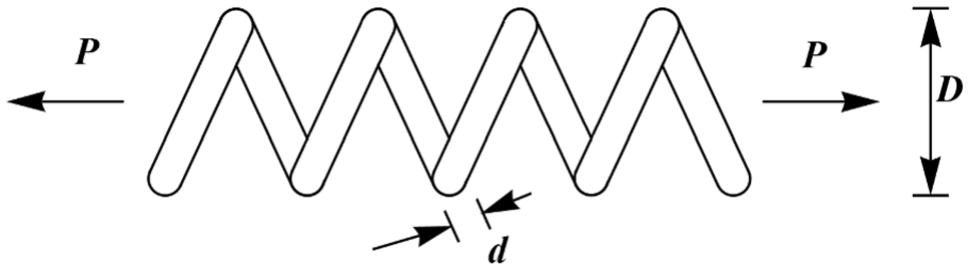
Schematics of the tension/compression spring design.

Tension/compression spring design is a real-world application aimed at minimizing tension/compression spring weight. The schematic of this design is presented in Fig. 11. The mathematical model of tension/compression spring is as follows^23^:

$$Consider:X=\left[{x}_{1}, {x}_{2}, {x}_{3} \right]=\left[d, D, P\right].$$

$$Minimize: f\left(x\right)=\left({x}_{3}+2\right){x}_{2}{x}_{1}^{2}.$$

$$Subject to:$$

$${g}_{1}\left(x\right)= 1-\frac{{x}_{2}^{3}{x}_{3}}{71785{x}_{1}^{4}} \le 0, {g}_{2}\left(x\right)=\frac{4{x}_{2}^{2}-{x}_{1}{x}_{2}}{12566({x}_{2}{x}_{1}^{3})}+\frac{1}{5108{x}_{1}^{2}}-1\le 0,$$

$${g}_{3}\left(x\right)= 1-\frac{140.45{x}_{1}}{{x}_{2}^{2}{x}_{3}}\le 0, {g}_{4}\left(x\right)=\frac{{x}_{1}+{x}_{2}}{1.5}-1 \le 0.$$

With

$$0.05\le {x}_{1}\le 2, {0.25\le x}_{2}\le 1.3 \mathrm{and} 2\le {x}_{3}\le 15.$$

**Figure 12:**
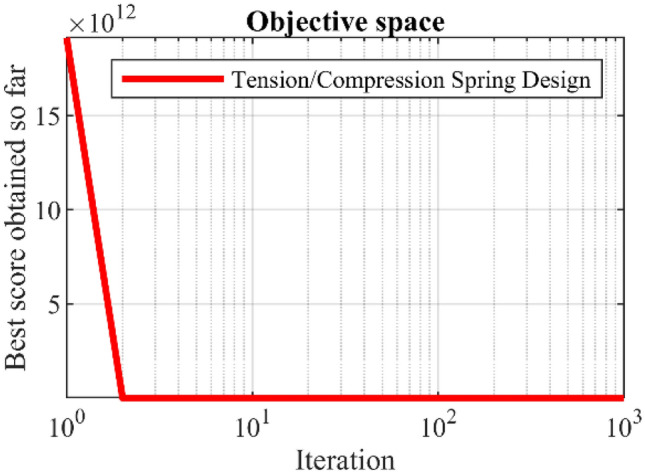
Convergence curves of PEOA on the tension/compression spring design.

The results of tension/compression spring optimization using PEOA and competitor algorithms are presented in Table 11. Based on the simulation results, PEOA has presented the optimal design of this problem with the values of the design variables equal to $(0.051606, 0.354725, 11.40679)$ and the objective function equal to $0.012665$. The convergence curve of PEOA in tension/compression spring optimization is presented in Fig. 12. Based on the analysis of simulation results, it is concluded that PEOA has provided more effective performance in tension/compression spring optimization compared to competitor algorithms.

**Table 11:** Evaluation results of the tension/compression spring design ($SIs$ are statistical indicators and $DVs$ are design variables).

		PEOA	RSA	MPA	TSA	WOA	GWO	MVO	TLBO	GSA	PSO	GA
$SIs$	Mean	0.01268	0.01966	0.01279	0.0131	0.01394	0.01759	0.01274	0.01857	0.01916	0.01781	2.8E+12
	Best	0.01266	0.01321	0.01279	0.0127	0.01267	0.01331	0.01272	0.01803	0.01396	0.01777	0.01804
	Worst	0.01272	0.04343	0.01279	0.01379	0.01777	0.01837	0.01287	0.01967	0.02484	0.01843	2.32E+13
	Std	2.0E−05	0.00999	7E−09	0.00033	0.00137	0.00126	4.03E−05	0.00043	0.00317	0.00015	6.16E+12
	Med	0.012668	0.01332	0.01279	0.013041	0.013692	0.017957	0.012729	0.018565	0.018669	0.017773	0.02599
	Rank	1	10	3	4	5	6	2	8	9	7	11
*DVs*	*D*	0.05161	0.05	0.05169	0.052014	0.052149	0.057255	0.05	0.069225	0.057216	0.068994	0.069257
	*D*	0.35472	0.310732	0.35671	0.36426	0.367878	0.505956	0.317411	0.940788	0.489072	0.933432	0.940437
	*P*	11.4068	15	11.2897	10.87649	10.66345	6.02195	14.03385	2	6.721057	2	2

## Conclusion and future works

In this paper, a new human-based metaheuristic algorithm called Preschool Education Optimization Algorithm (PEOA) was introduced. The design of PEOA draws its primary inspiration from the dynamic processes within preschool education. The proposed PEOA was explained in three phases (i) the gradual growth of the preschool teacher's educational influence, (ii) individual knowledge development guided by the teacher, and (iii) individual increase of knowledge and self-awareness, and then its mathematical model was presented. A set of fifty-two standard benchmark functions representing unimodal, high-dimensional multimodal, fixed-dimensional multimodal, and CEC 2017 test suite were utilized to assess the optimization capabilities of PEOA. The obtained optimization results highlighted PEOA's proficiency in effectively balancing global exploration and local exploitation during the search. Additionally, PEOA's performance was benchmarked against ten established metaheuristic algorithms. Analyzing the simulation outcomes revealed that PEOA consistently outperforms competing algorithms by delivering enhanced solutions across a majority of benchmark functions. Furthermore, when applied to address twenty-two real-world optimization problems from CEC 2011 test suite and four distinct engineering design challenges, PEOA demonstrated remarkable efficacy in resolving real-world problems, underscoring its efficiency in practical applications.

The proposed PEOA approach has several advantages for global optimization problems. Against these advantages, PEOA also has several disadvantages. The main advantage of PEOA is that its mathematical model has no control parameters, which must be adjusted by the user (of course, exclude the population size $N$ and maximal number of iterations $T$). For this reason, the proposed approach does not need the parameter tuning process. The second advantage of PEOA is its high efficiency in dealing with various optimization problems in many sciences and complex high-dimensional problems. The proposed method's third advantage is its excellent ability to balance exploration and exploitation in the search process, which allows high-speed convergence to provide suitable values for decision variables in optimization tasks, especially in complex problems. The fourth advantage of the proposed PEOA is its robust performance in handling real-world optimization applications. The proposed PEOA approach is a stochastic-based solving method. So, the main disadvantage of PEOA, similar to all stochastic-based optimizers, is there is no guarantee that PEOA will achieve the global optimal solution. In addition, PEOA may fail to address some optimization applications because, according to the NFL theorem, there is no a priory presumption that any metaheuristic algorithm will be successful or not. Another disadvantage of PEOA is that it is always possible to develop newer algorithms that perform better than existing algorithms and PEOA.

The introduction of the PEOA approach paves the way for many potential avenues of future investigation and development. One notable prospect is the development of binary and multi-objective adaptations of the PEOA, which holds significant promise for further research. Additionally, exploring the application of PEOA in tackling optimization challenges across diverse disciplines and real-world scenarios offers an enticing direction for future studies.

### Ethical approval

This article does not contain any studies with human participants or animals performed by the author.

### Informed consent

Informed consent was not required as no human or animals were involved.

## Data Availability

All data generated or analyzed during this study are included directly in the text of this submitted manuscript. There are no additional external files with datasets.
